# **α**7nAChR activation in AT2 cells promotes alveolar regeneration through WNT7B signaling in acute lung injury

**DOI:** 10.1172/jci.insight.162547

**Published:** 2023-08-08

**Authors:** Xiaoyan Chen, Cuiping Zhang, Tianchang Wei, Jie Chen, Ting Pan, Miao Li, Lu Wang, Juan Song, Cuicui Chen, Yan Zhang, Yuanlin Song, Xiao Su

**Affiliations:** 1 Shanghai Key Laboratory of Lung Inflammation and Injury, Department of Pulmonary Medicine, Zhongshan Hospital, Fudan University, Shanghai, China.; 2 Unit of Respiratory Infection and Immunity, Chinese Academy of Sciences, Key Laboratory of Molecular Virology and Immunology, Institut Pasteur of Shanghai, Chinese Academy of Sciences, Shanghai, China.; 3 Department of Hematology, Shanghai General Hospital, Shanghai Jiaotong University School of Medicine, Shanghai, China.; 4 Shanghai Institute of Infectious Disease and Biosecurity, Shanghai, China.; 5 Shanghai Respiratory Research Institute, Shanghai, China.; 6 National Clinical Research Center for Aging and Medicine, Huashan Hospital, Fudan University, Shanghai, China.; 7 Jinshan Hospital of Fudan University, Shanghai, China.

**Keywords:** Pulmonology, Stem cells, Adult stem cells, Respiration

## Abstract

Reducing inflammatory damage and improving alveolar epithelium regeneration are two key approaches to promoting lung repair in acute lung injury/acute respiratory distress syndrome (ALI/ARDS). Stimulation of cholinergic α7 nicotinic acetylcholine receptor (α7nAChR, coded by *Chrna7*) signaling could dampen lung inflammatory injury. However, whether activation of α7nAChR in alveolar type II (AT2) cells promotes alveolar epithelial injury repair and underlying mechanisms is elusive. Here, we found that α7nAChR was expressed on AT2 cells and was upregulated in response to LPS-induced ALI. Meanwhile, deletion of *Chrna7* in AT2 cells impeded lung repair process and worsened lung inflammation in ALI. Using in vivo AT2 lineage–labeled mice and ex vivo AT2 cell–derived alveolar organoids, we demonstrated that activation of α7nAChR expressed on AT2 cells improved alveolar regeneration by promoting AT2 cells to proliferate and subsequently differentiate toward alveolar type I cells. Then, we screened out the WNT7B signaling pathway by the RNA-Seq analysis of in vivo AT2 lineage–labeled cells and further confirmed its indispensability for α7nAChR activation–mediated alveolar epithelial proliferation and differentiation. Thus, we have identified a potentially unrecognized pathway in which cholinergic α7nAChR signaling determines alveolar regeneration and repair, which might provide us a novel therapeutic target for combating ALI.

## Graphical abstract

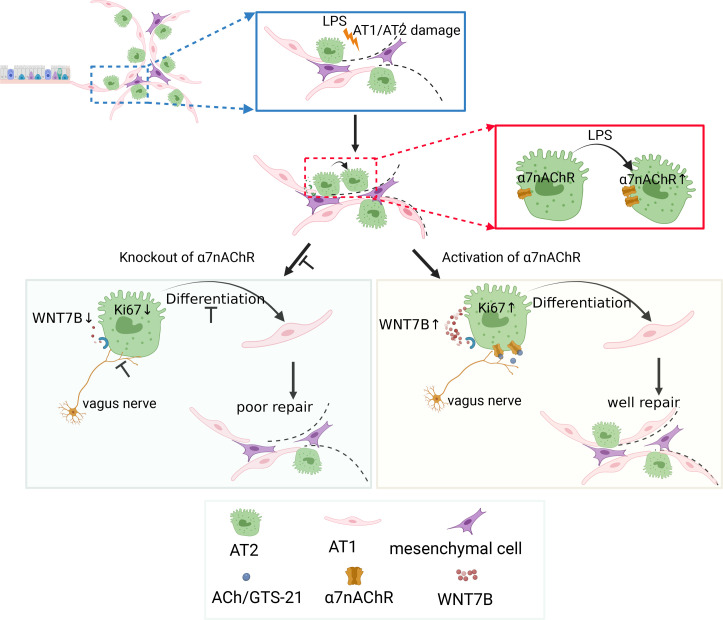

## Introduction

Acute diffuse alveolar injury, which is characterized by accumulation of massive inflammatory cytokines and immune cells and alveolar epithelium damage in the alveoli, makes up the basic pathology of acute lung injury/acute respiratory distress syndrome (ALI/ARDS) (1). Thus, therapeutic approaches that can both reduce inflammation and promote alveolar epithelium/capillary endothelium regeneration may alleviate lung injury and improve patient outcomes.

The α7 nicotinic acetylcholine receptor (α7nAChR), a homomeric calcium channel that is expressed not only in nervous systems but also other nonneuronal tissues, senses vagal signals via the parasympathetic neurotransmitter acetylcholine (2) and has been reported as a potential therapeutic target in neurodegenerative (3), neuropsychiatric (4), and inflammatory disease (2, 5, 6). In pulmonary disease, α7nAChR has been shown to attenuate lung inflammation and tissue damage in *E*. *coli*–, LPS- (7), Gram-negative sepsis– (8), acid- (9), and radiation-induced (10) lung injury and repress ILC2-dependent airway hyperreactivity (11). In addition to cholinergic antiinflammatory effects, evidence supports that α7nAChR is involved in regulating the proliferative (12, 13), differentiative (14, 15), and cell migration capacity (12) of stem cells and their stem cell niche (14) in different organs. Emerging data show that α7nAChR has been involved in several vital biological processes in the lung, such as cell proliferation, angiogenesis, and apoptosis in cancer (16, 17). Prenatal exposure to nicotine upregulates pulmonary α7nAChR expression and alters fetal lung development (18), subsequently adversely affecting pulmonary function at birth (19). In particular, nicotine exposure alters lung branching morphogenesis via α7nAChR (20). α7nAChR is a key regulator of the plasticity of the airway epithelium through control of basal cell proliferation and differentiation (21). Altogether, these studies led us to investigate whether α7nAChR could be involved in alveolar regeneration.

Alveolar epithelium is especially vulnerable to injury because its surface is constantly and directly exposed to various external environments (1). The reparative process of the alveolar epithelium relies on the proliferation of alveolar type II (AT2) cells, followed by differentiation into alveolar type I (AT1) cells (22, 23). Thus, a quick and effective response of AT2 cells after lung injury guarantees normal reparative process and function of alveoli. Upon alveolar epithelium injury, normally quiescent AT2 cells are quickly activated and recruited to act as progenitor cells to replenish AT1 cells and repair alveolar epithelium (24). Unlike rare AT2 stem cells during homeostasis, which are regulated by the fibroblast-juxtacrine wingless-type MMTV integration site family (WNT) niche, these cells transiently activate autocrine secretion of WNT, such as WNT7B (24). Nonetheless, the precise mechanism for triggering a quick WNT secretion of AT2 cells is poorly understood.

Here, we planned to investigate whether and how α7nAChR regulates the cellular identity of AT2 cells during alveolar regeneration after injury. We demonstrated that activation of α7nAChR expressed on AT2 cells improved alveolar regeneration by promoting AT2 cells to proliferate and subsequently differentiate toward AT1 cells. Moreover, RNA-Seq analysis of in vivo AT2 lineage–labeled cells allowed us to screen out the WNT7B signaling pathway that is requisite for the α7nAChR activation–mediated alveolar regeneration process. These potentially novel findings provide us insights into how vagal α7nAChR signaling promotes alveolar injury repair.

## Results

### LPS upregulates the expression of α7nAChR on AT2 cells.

**Figure 1:**
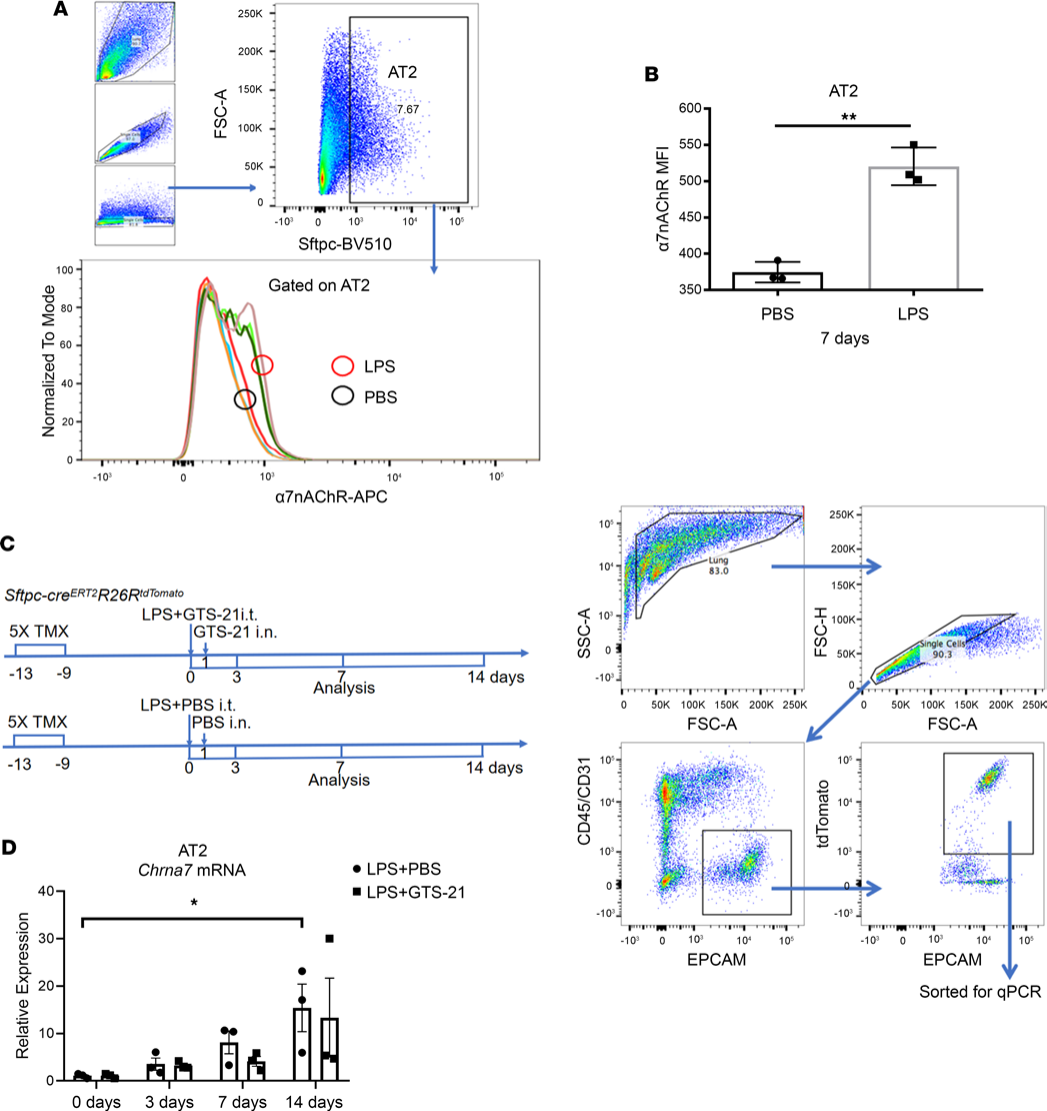
LPS upregulates the expression of α7nAChR on AT2 cells. (**A**) The flow cytometric gating strategy for detecting the α7 nicotinic acetylcholine receptor (α7nAChR) protein on alveolar type II (AT2) cells using fluorochrome-conjugated α-bungarotoxin, a nicotinic cholinergic blocker. PBS or LPS (2.5 mg/kg) was intratracheally delivered to mice and was followed for 7 days. As shown in the Figure, AT2 cells account for about 7.67% in all lung cells in this indicated sample. (**B**) Median fluorescence intensity (MFI) of α7nAChR protein on AT2 cells (2-sided *t* test). (**C**) Intervention schematic diagram and sorting strategy for lineage-labeled AT2 cells by flow cytometry at indicated time points. PBS, LPS (2.5 mg/kg), or GTS-21 (α7nAChR selective agonist, 4 mg/kg) was intratracheally delivered to lineage-tracing mice (*Sftpc-cre^ERT2^R26R^tdTomato^*), followed by intranasal administration of PBS or GTS-21 on day 1. Lineage-labeled AT2 cells were sorted, and the expression of α7nAChR *(Chrna7*) was quantified at the mRNA level by quantitative real-time PCR (qPCR) at the 0th, 3rd, 7th, and 14th day. Specific time points for tamoxifen (TMX) injection are indicated. (**D**) *Chrna7* gene expression in AT2 cells (1-way ANOVA with Tukey’s post hoc analysis). Data are representative of at least 3 independent experiments and are presented as mean ± SD (*N* = 3; *P < 0.05, **P < 0.01).

It has been reported that α7nAChR was presented on inflammatory cells (2, 7), alveolar macrophages, club cells, and AT2 cells (25), and CD45^–^ α7^lineage+^ populations in lung contained 79% epithelial cells (26). However, whether α7nAChR expressed on AT2 cells responds to LPS-induced lung injury is still unknown. In our study, we found that AT2 cells expressed α7nAChR and that its expression was significantly upregulated in mice treated with LPS, when compared with mice in the PBS-treated control group (Figure 1, A and B). This result was further confirmed by AT2 cell sorting and quantitative real-time PCR (qPCR) experiments (Figure 1C), in which LPS challenge augmented the α7nAChR gene expression of AT2 cells in a time-dependent manner (Figure 1D). However, a selective α7nAChR agonist (GTS-21) did not change the gene expression of α7nAChR in AT2 cells (Figure 1D). Taken together, these results indicate that AT2 cells expressed α7nAChR in response to LPS challenge.

### Deletion of Chrna7 on AT2 cells impedes the lung repair process and increases lung proinflammatory responses after lung injury.

**Figure 2:**
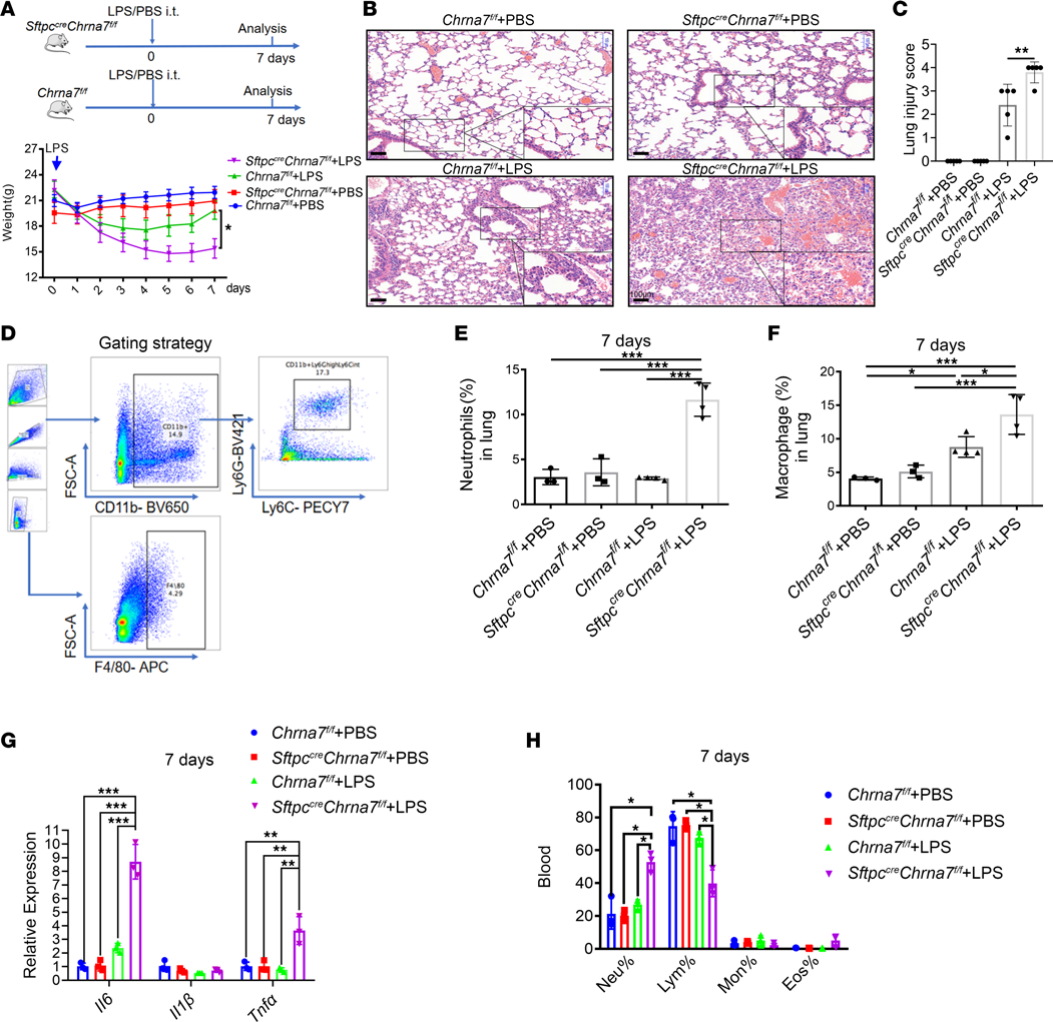
Deletion of *Chrna7* on AT2 cells impedes the lung repair process and increases lung proinflammatory responses after lung injury. (**A**) PBS or LPS (2.5 mg/kg) was intratracheally (i.t.) delivered to *Sftpc^cre^Chrna7^fl/fl^* mice or *Chrna7^fl/fl^* mice and was followed for 7 days. Changes in mice body weight in the *Chrna7^fl/fl^*+PBS, *Chrna7^fl/fl^*+LPS, *Sftpc^cre^Chrna7^fl/fl^*+PBS, or *Sftpc^cre^Chrna7^fl/fl^*+LPS groups (2-way ANOVA with Šidák’s post hoc analysis). (**B**) Representative H&E-stained lung sections on day 7 after injury. Insets show high-power images. Scale bars: 100 μm. (**C**) Lung injury score. (**D**) Flow cytometric gating strategy for neutrophils and macrophages in lung. (**E**) The percentage of neutrophils (CD11b^+^Ly6G^hi^Ly6C^intermediate^ cells) in lung was determined by flow cytometry on day 7 after injury. (**F**) The percentage of macrophages (F4/80^+^ cells) in lung was determined by flow cytometry on day 7 after injury. (**G**) The relative gene expression of *Il6*, *Il1*β, and *Tnfα* in lung tissue homogenate was tested by qPCR on day 7 after injury. (**H**) The percentage of neutrophils (Neu), lymphocytes (Lym), monocytes (Mon), and eosinophils (Eos) in the blood was determined using a multispecies hematology instrument on day 7 after injury. One-way ANOVA with Tukey’s post hoc analysis was used in **C** and **E**–**H**. Data are representative of at least 3 independent experiments and are presented as mean ± SD (*N* = 3–5; *P < 0.05, **P < 0.01, ***P < 0.001).

To assess whether the previously observed LPS-induced α7nAChR expression in AT2 cells is involved in the lung repair process after injury, we generated *Sftpc^cre^Chrna7^fl/fl^* mice to deplete α7nAChR specifically in AT2 cells (Supplemental Figure 1A; supplemental material available online with this article; https://doi.org/10.1172/jci.insight.162547DS1). Then, we challenged *Sftpc^cre^Chrna7^fl/fl^* mice and control mice with PBS or LPS. As shown in Figure 2A, deletion of *Chrna7* on AT2 cells significantly decreased mouse body weight and delayed mouse recovery and impeded the lung repair process (including reduced fluid exudation, inflammatory cell infiltrate, vasodilation, and recovery of normal lung structure, Figure 2, B and C). In addition, the infiltrate of neutrophils (CD11b^+^Ly6G^hi^Ly6C^intermediate^ cells) and macrophages (F4/80^+^ cells) in lung significantly increased in *Sftpc^cre^Chrna7^fl/fl^* mice treated with LPS, compared with that in *Chrna7^fl/fl^* mice treated with LPS (Figure 2, D–F). Accordingly, the gene expression of inflammatory cytokines (*Il6* and *Tnfα*) in *Sftpc^cre^Chrna7^fl/fl^* mice treated with LPS was much higher than that in *Chrna7^fl/fl^* mice treated with LPS (Figure 2G). Meanwhile, deletion of *Chrna7* in AT2 cells led to an increase of neutrophils in the blood but a decrease of lymphocytes in blood (Figure 2H). These results suggest that α7nAChR expression in AT2 cells is implicated in the lung repair process after injury.

### Activation of α7nAChR on AT2 cells is required for alveolar regeneration.

**Figure 3:**
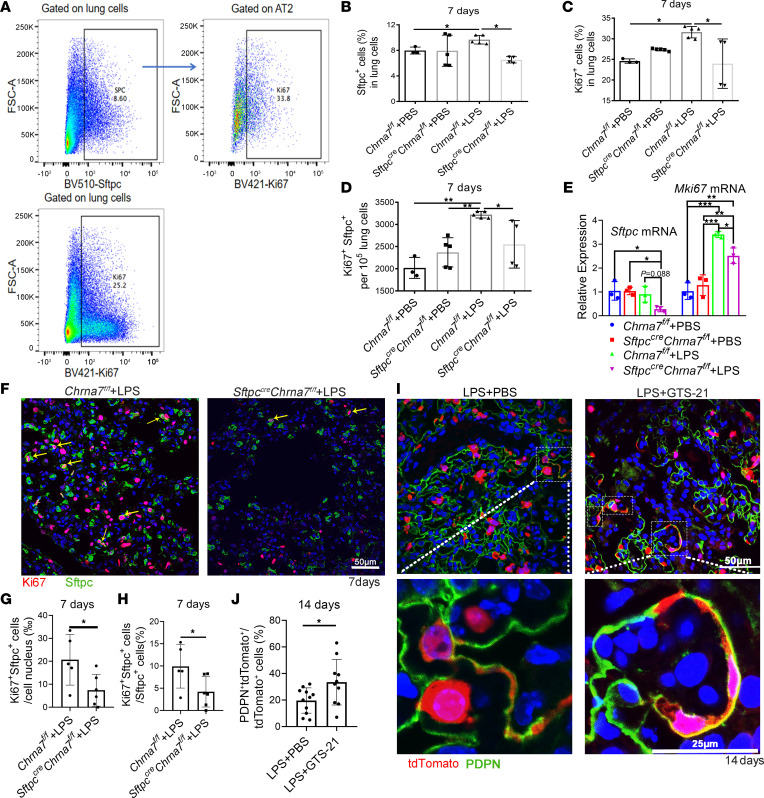
α7nAChR expressed on AT2 cells is required for alveolar regeneration in vivo. (**A**) PBS or LPS (2.5 mg/kg) was intratracheally delivered to *Sftpc^cre^Chrna7^fl/fl^* mice or *Chrna7^fl/fl^* mice and was followed for 7 days. The flow cytometric gating strategy for detecting Sftpc^+^ cells (AT2 cells), Ki67^+^ cells (proliferating cells), and Sftpc^+^Ki67^+^ cells (proliferating AT2 cells). (**B**) The percentage of Sftpc^+^ cells in mouse lung. (**C**) The percentage of Ki67^+^ cells in mouse lung. (**D**) The number of Sftpc^+^Ki67^+^ cells per 10^5^ lung cells. (**E**) The relative gene expression of *Sftpc* and *Mki67* in lung tissue homogenate was tested by qPCR. (**F**) Representative immunofluorescence (IF) images showing Sftpc^+^ cells, Ki67^+^ cells, and Sftpc^+^Ki67^+^ cells in the lungs of *Sftpc^cre^Chrna7^/f^* mice treated with LPS or Chrna7*^fl/fl^* mice treated with LPS on day 7 after injury (Ki67, red; Sftpc, green; DAPI, blue). Arrows indicate Sftpc^+^Ki67^+^ cells. Scale bars: 50 μm. (**G** and **H**) Quantification of Ki67^+^Sftpc^+^ cells in **F**. Each individual dot represents 1 section. (**I**) Representative IF images showing podoplanin^+^ (PDPN^+^) AT1 cell differentiation from *Sftpc* lineage–labeled cells on day 14 after injury in the lungs of indicated groups (tdTomato, red; PDPN, green; DAPI, blue). Scale bars: 50 μm. (**J**) Quantification of lineage-labeled PDPN^+^ AT1 cells in **H**. Each individual dot represents 1 section. One-way ANOVA with Tukey’s post hoc analysis was used in **B**–**E**. Two-sided *t* test was used in **G**, **H**, and **J**. Data are representative of at least 3 independent experiments and are presented as mean ± SD (*N* = 3–5; *P < 0.05, **P < 0.01, ***P < 0.001).

Given that knockout of α7nAChR in AT2 cells impedes the recovery of lung structure, we next asked whether α7nAChR expressed on AT2 cells is required for alveolar regeneration. To answer this question, we detected the changes in proliferative and differentiative properties of AT2 cells. As expected, we discovered that the percentage of Sftpc^+^ cells (AT2 cells) and Ki67^+^ cells (proliferating cells) and the number of Sftpc^+^Ki67^+^ cells (proliferating AT2 cells) in the lungs of LPS-treated *Sftpc^cre^ Chrna7^fl/fl^* mice significantly decreased, when compared with those in the lungs of LPS-treated *Chrna7^fl/fl^* mice on day 7 (Figure 3, A–D). qPCR analysis revealed a decreased trend of *Sftpc* gene expression and significantly reduced *Mki67* gene expression in the lungs of LPS-treated *Sftpc^cre^Chrna7^fl/fl^* mice relative to that in the lungs of LPS-treated *Chrna7^fl/fl^* mice (Figure 3E). Consistent with the flow cytometry results, a discernible difference in the number of Ki67^+^ AT2 cells was observed in the immunofluorescence-stained lungs of indicated genotyped mice (Figure 3, F–H), indicating that deletion of *Chrna7* in AT2 cells impairs the proliferative properties of AT2 cells. However, the number of podoplanin^+^ (PDPN^+^) cells (AT1 cells) and CCSP^+^ cells (club cells) was not reduced in the lungs of *Sftpc^cre^Chrna7^fl/fl^* mice on day 7 after injury (Supplemental Figure 1, B–E). In the in vitro study, activation of α7nAChR in AT2 cells led to a significantly increased expression of *Pdpn* and *Mki67* on day 5 after GTS-21 (selective α7nAChR agonist) treatment, which could be blocked by MLA (α7nAChR antagonist, Supplemental Figure 2). To elucidate whether α7nAChR activation could drive AT2-to-AT1 differentiation in vivo, we used a LPS-induced ALI model in AT2 lineage–tracing mice and found a significant increase in the number of lineage-labeled AT1 cells in the lungs of α7nAChR agonist–treated mice on day 14 compared with that in the lungs of PBS-treated mice (Figure 3, I and J, and Supplemental Figure 3, A and B).

**Figure 4:**
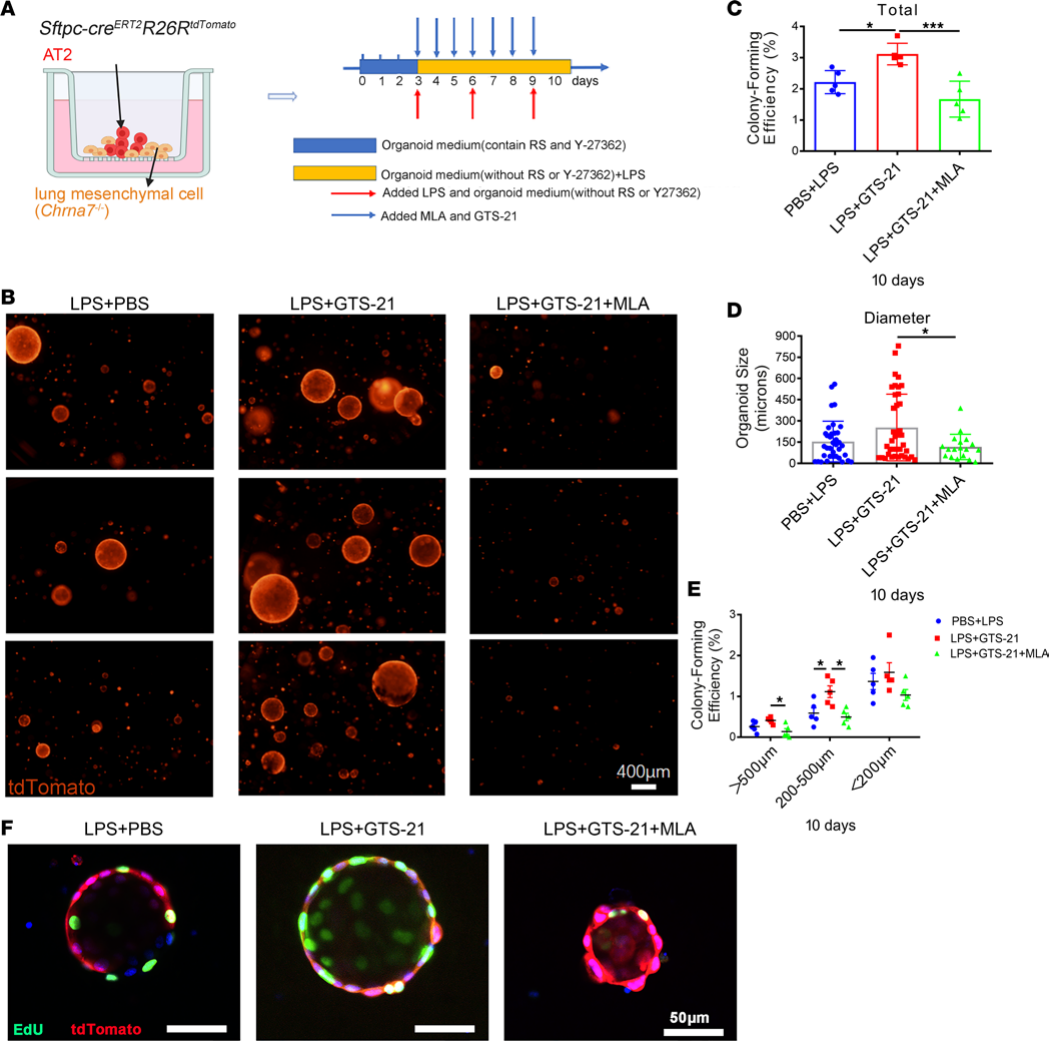
Activation of α7nAChR on AT2 cells promotes alveolar organoid formation. (**A**) Schematic of organoid coculture of *Sftpc* lineage–labeled cells (CD45^–^CD31^–^EPCAM^+^tdTomato^+^) with lung mesenchymal cells (CD45^–^CD31^–^EPCAM^–^) isolated from α7nAChR-knockout (*Chrna7^–/–^*) mice and the intervention diagram; LPS (1 μg/mL) was added to simulate lung injury in vitro and methyllycaconitine citrate (MLA; α7nAChR antagonist, 10 μmol/L) 15 minutes before GTS-21 (selective α7nAChR agonist, 10 μmol/L) treatment. Times for renewing organoid medium and adding LPS, GTS-21, and MLA are indicated. The culture time varies in different experiments according to purpose. RS, R-Spondin-1. (**B**) Representative fluorescence images of AT2 organoids captured on day 10. Scale bars: 400 μm. (**C**) Statistical quantification of the total colony formation efficiency of alveolar organoids. Each individual dot represents 1 experiment in 1 mouse. (**D**) Statistical quantification of the size of alveolar organoids. Each individual dot represents 1 organoid. (**E**) Statistical quantification of total colony formation efficiency of alveolar organoids of different sizes. (**F**) Representative fluorescence images showing proliferating cells in AT2 organoids derived from the lungs of lineage-tracing mice. Organoids were treated with 5-ethynyl-2′-deoxyuridine (EdU) at an early time point (day 8) for 3 hours in cultures (tdTomato, red; EdU, green; DAPI, blue). Scale bars: 50 μm. One-way ANOVA with Tukey’s post hoc analysis was used in **C**–**E**. Data are representative of at least 3 independent experiments and are presented as mean ± SD (*P < 0.05, ***P < 0.001).

For confirmation, we utilized a 3D organoid model and found fewer lung organoids in *Sftpc*^cre^*Chrna7^fl/fl^* mice compared with *Chrna7^fl/fl^* mice after injury and that α7nAChR agonist could not reverse this change (Supplemental Figure 4A). To exclude the interference from lung mesenchymal cells and other lung stem cells, we further created 3D organoids by coculturing lineage-labeled AT2 cells with lung mesenchymal cells from *Chrna7*-knockout mice. Of note, alveolar organoids in the presence of GTS-21 were more numerous and formed a larger alveolar organoid formation than those without GTS-21; however, this was reversed by MLA pretreatment (Figure 4, A–E, and Supplemental Figure 4B). Similarly, we observed a significantly higher colony-forming efficiency in the PNU-282987–treated group (Supplemental Figure 5, A–D). In addition, assessing proliferation with 5-ethynyl-2′-deoxyuridine (EdU) incorporation assays at an early time point (day 8), we found that α7nAChR agonist treatment enhanced proliferative capacity of AT2 lineage–labeled cells, but MLA pretreatment blocked this effect (Figure 4F and Supplemental Figure 5E). This result was further supported by the qPCR assay in the AT2 cell in vitro culture (Supplemental Figure 2B). Overall, these findings suggest that activation of α7nAChR expressed on AT2 cells promotes AT2 cells to proliferate and, ultimately, differentiate to AT1 cells, which contributes to alveolar regeneration.

### Vagal α7nAChR signaling upregulates canonical WNT7B signaling in AT2 cells during LPS-induced lung injury.

**Figure 5:**
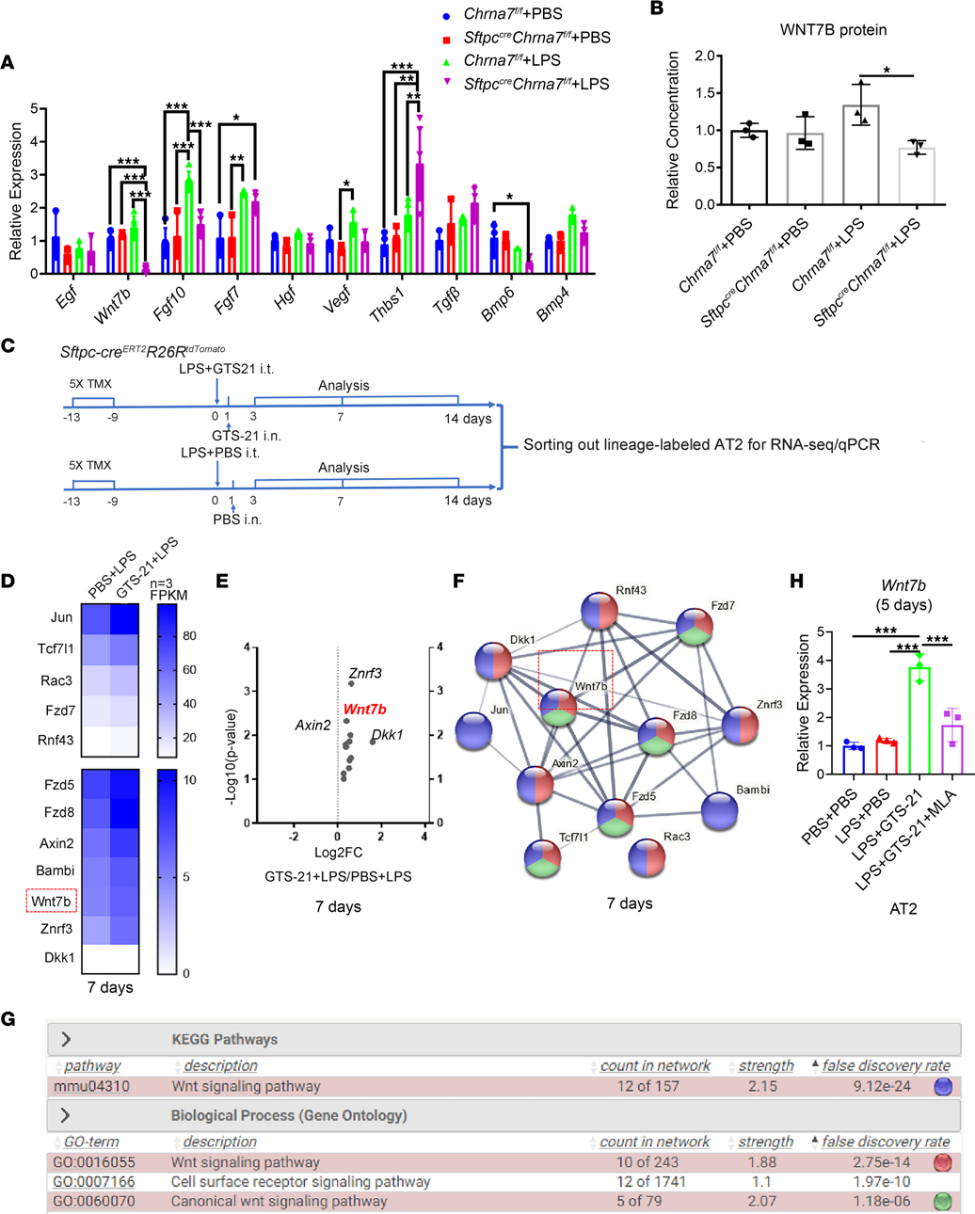
Vagal-7nAChR signaling upregulates canonical WNT7B signaling in AT2 cells during LPS-induced lung injury. (**A** and **B**) PBS or LPS (2.5 mg/kg) was intratracheally delivered to *Sftpc^cre^Chrna7^fl/fl^* mice or *Chrna7^fl/fl^* mice and was followed for 7 days (*N* = 3–5 in each group). (**A**) qPCR analysis of genes that are key growth factors for lung regeneration in the lung tissue. (**B**) Statistical quantification of the relative concentration of wingless-type MMTV integration site family, member 7B (WNT7B) protein in the lung tissue tested by ELISA. (**C**) PBS, LPS (2.5 mg/kg), or GTS-21 (α7nAChR selective agonist, 4 mg/kg) was intratracheally (i.t.) delivered to lineage-tracing mice (*Sftpc-cre^ERT2^R26R^tdTomato^*) followed by PBS or GTS-21 intranasal administration (i.n.) at the 1st day, and *Sftpc* lineage–labeled cells were sorted for RNA-Seq and qPCR analysis. Specific time points for tamoxifen (TMX) injection and analysis are indicated (*N* = 3 in each group). (**D**) Heatmap of the transcriptional profiles of *Wnt7b* and other related genes in AT2 cells on day 7. (**E**) Volcano plot analysis of gene changes presented in **D**. (**F**) Protein interaction network among genes presented in **D**. (**G**) KEGG pathway and GO analysis of *Wnt7b* and other related genes presented in **D**. (**H**) AT2 cells were purified by FACS and then treated with PBS, LPS (1 μg/mL), GTS-21 (10 μmol/L), or MLA (10 μmol/L) in vitro, and the gene expression of *Wnt7b* was quantified by qPCR on day 5. One-way ANOVA with Tukey’s post hoc analysis was used in **A**, **B**, and **H**. Data are representative of at least 3 independent experiments and are presented as mean ± SD (*P < 0.05, **P < 0.01, ***P < 0.001).

Growth factor–mediated signaling pathways are essential for lung homeostasis of progenitor AT2 stem cell function (27). To find out how vagal α7nAChR signaling affects the behavior of AT2 cells, we performed qPCR to screen out the key molecules, including *Egf,* *Fgf7*, *Fgf10*, *Hgf*, *Vegf*, *Thbs1*, *Tgfβ*, *Wnt7b*, *Bmp4*, and *Bmp6*. Remarkably, *Wnt7b* gene expression, mainly expressed in AT2 cells (24), was significantly downregulated in the lungs of Sftpc^cre^*Chrna7^fl/fl^* mice treated with LPS, when compared with that of *Chrna7^fl/fl^* mice treated with LPS (Figure 5A). Consistently, the ELISA experiment showed an increased presense of WNT7B protein in the lung of *Chrna7^fl/fl^* mice treated with LPS relative to those without LPS treatment and a significant decrease of WNT7B protein in the lungs of *Sftpc^cre^Chrna7^fl/fl^* mice treated with LPS relative to that of *Chrna7^fl/fl^* mice treated with LPS (Figure 5B). Considering that WNT7B is exclusively expressed in the lung epithelium, including airway epithelium (28) and alveolar epithelium (24), we meticulously removed trachea before homogenizing lung tissue in our experiments; thus, the WNT7B expression changes can be considered to be predominately contributed by alveolar epithelium.

Furthermore, RNA-Seq analysis was performed on FACS-purified lineage-labeled AT2 cells from both PBS- and GTS-21–treated *Sftpc* lineage–tracing mice after LPS challenge (Figure 5C). By analyzing the top most expressed genes, we found that AT2 cell–specific genes, such as *Sftpc* and *Sftpb*, were enriched in sorted cells (Supplemental Figure 6, A–C). On day 3, *Wnt7b* was upregulated in the GTS-21+LPS group compared with the PBS+LPS group (Supplemental Figure 7, A–C). On day 7, *Wnt7b* and other related genes, *Axin2*, *Dkk1*, *Fzd5*, *Fzd7*, and *Fzd8*, were increased in the GTS-21+LPS group compared with the PBS+LPS group (Figure 5, D–F), indicating that WNT7B signaling is activated at this stage (Figure 5G). On day 14, the upregulated genes were enriched in nucleosomal DNA binding, chromatin DNA binding, and circadian rhythm, indicating that the reparative process was under way (Supplemental Figure 8, A–C). We used qPCR to further confirm an increased expression of *Wnt7b* in FACS-sorted AT2 cells from GTS-21–treated mice after LPS insult on day 7 compared with control mice (Supplemental Figure 9A). Correspondingly, in the in vitro study, we observed a remarkably increased expression of *Wnt7b* in AT2 cells after GTS-21 treatment on day 5 after LPS challenge, which was significantly reversed by MLA (Figure 5H). In the LPS-challenged vagotomized lungs, WNT7B expression at both mRNA and protein levels was reduced compared with that in the LPS-challenged sham lungs (Supplemental Figure 9, B and C). At the protein level, this phenomenon was reversed by administrating GTS-21 in the LPS-challenged vagotomized lungs (Supplemental Figure 9D). These findings strongly support that vagal α7nAChR signaling is essential for upregulating WNT7B signaling in AT2 cells during lung injury repair.

### α7nAChR-driven WNT7B signaling is integral for α7nAChR-promoted alveolar regeneration.

**Figure 6:**
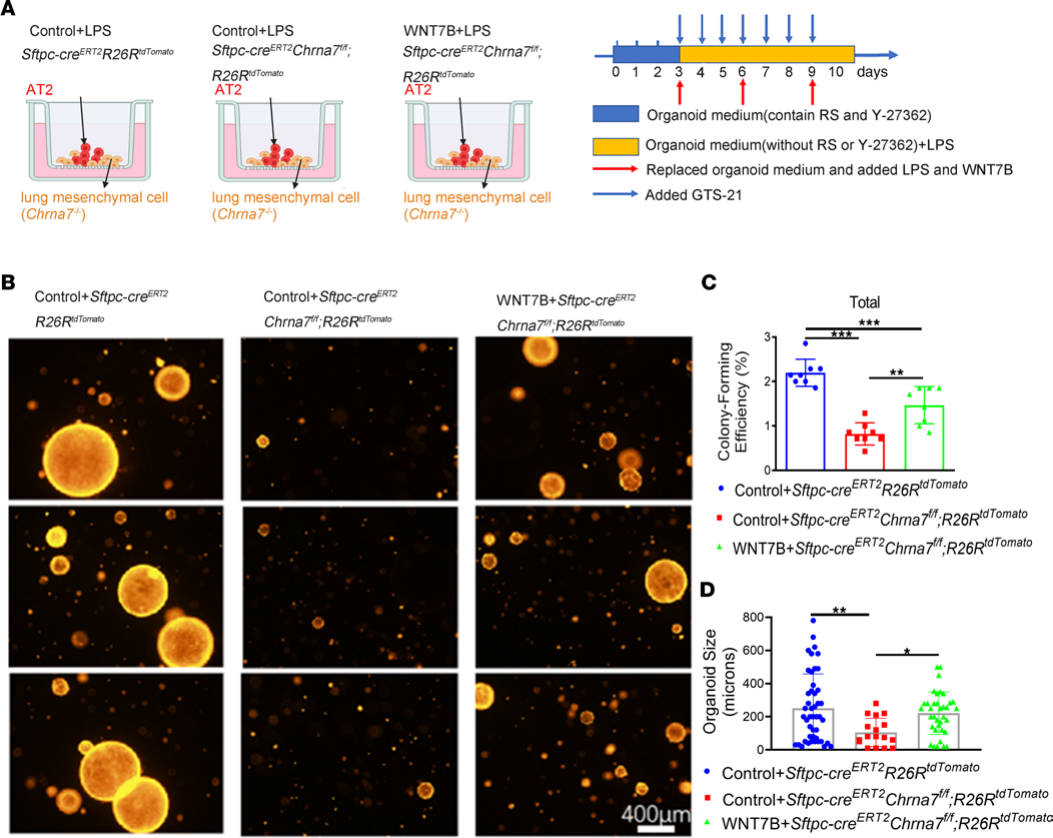
α7nAChR-driven WNT7B signaling is an essential mediator of α7nAChR-promoted alveolar organoid formation. (**A**) Schematic of organoid coculture of *Sftpc* lineage–labeled AT2 cells (CD45^–^CD31^–^EPCAM^+^tdTomato^+^) isolated from indicated mice, with lung mesenchymal cells (CD45^–^CD31^–^EPCAM^–^) isolated from α7nAChR-knockout (*Chrna7^–/–^*) mice, and the intervention diagram. (**B**) Representative IF images of AT2 organoids. LPS (1 μg/mL) was added to simulate lung injury in vitro, and GTS-21(10 μmol/L) was used to activate α7nAChR in all groups. WNT7B (100 ng/mL) was added as indicated. Scale bars: 400 μm. (**C**) Statistical quantification of the total colony formation efficiency of alveolar organoids. Each individual dot represents 1 experiment. (**D**) Statistical quantification of the size of alveolar organoids. Each individual dot represents 1 organoid. One-way ANOVA with Tukey’s post hoc analysis was used in **C** and **D**. Data are representative of at least 3 independent experiments and are presented as mean ± SD (*P < 0.05, **P < 0.01, ***P < 0.001).

Given that α7nAChR activation increased the generation of alveolar organoids and sustained a high WNT7B secretion in AT2 cells, we next asked whether WNT7B signaling is requisite for α7nAChR-promoted alveolar regeneration. To answer this question, we specifically deleted *Chrna7* in AT2 cells in *Sftpc* lineage–tracing mice (*Sftpc-cre^ERT2^Chrna7^fl/fl^R26R^tdTomato^*) and then lineage-labeled AT2 cells were sorted out for organoid culture and in vitro intervention (Figure 6A). Importantly, we discovered that *Chrna7* knockout of AT2 cells led to a remarkable decrease in the number and size of alveolar organoids relative to control cells. Meanwhile, supplement with WNT7B partly restored the colony-forming efficiency and size of alveolar organoids (Figure 6, B–D, and Supplemental Figure 4B).

**Figure 7:**
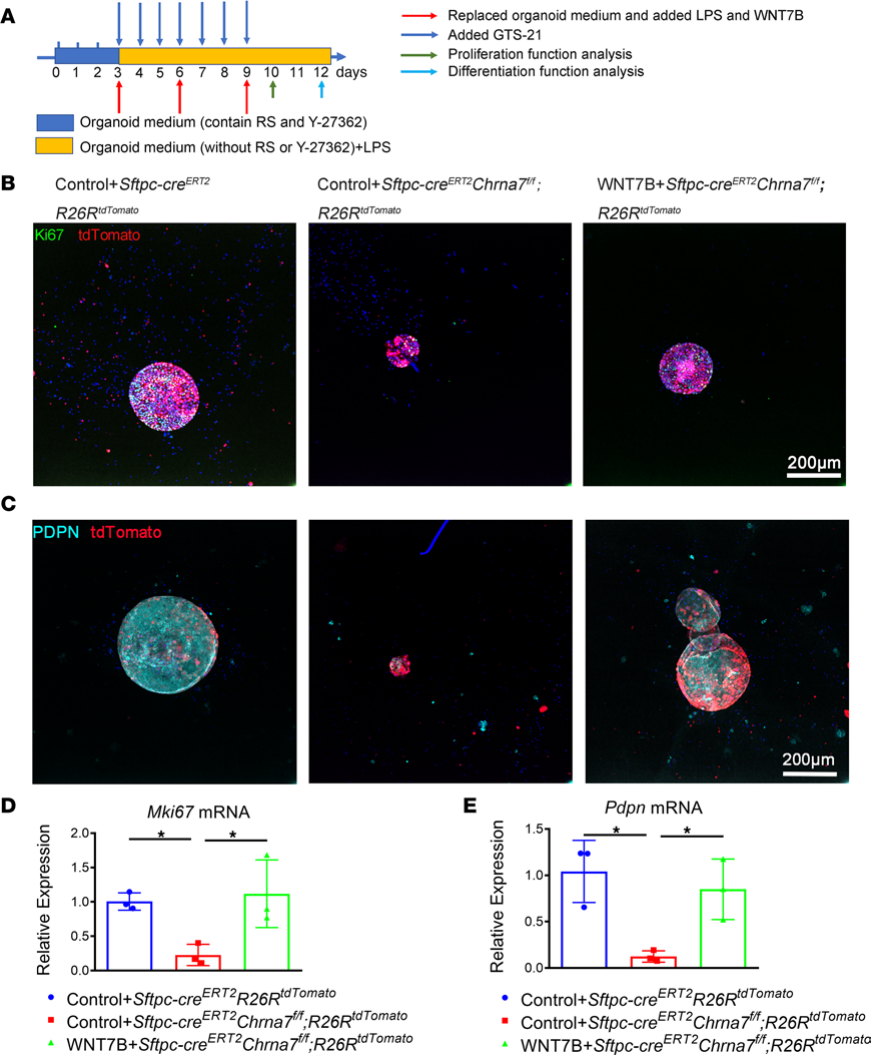
α7nAChR-driven WNT7B signaling is integral for α7nAChR-promoted AT2 cell proliferation and differentiation. (**A**) Schematic of organoid coculture of *Sftpc* lineage–labeled cells (CD45^–^CD31^–^EPCAM^+^tdTomato^+^) isolated from indicated mice, with lung mesenchymal cells (CD45^–^CD31^–^EPCAM^–^) isolated from α7nAChR-knockout (*Chrna7^–/–^*) mice, and the intervention diagram. (**B**) Effect of selective knockout of α7nAChR on AT2 cells and supplementation of WNT7B (100 ng/mL) on the proliferation of lineage-traced AT2 cells in organoids on day 10, as judged by Ki67 (proliferative marker) (tdTomato, red; Ki67, green; DAPI, blue). Scale bars: 200 μm. (**C**) Effect of selective knockout of α7nAChR on AT2 cells and supplementation of WNT7B (100 ng/mL) on the differentiation of lineage-traced AT2 cells in organoids on day 12, as judged by PDPN (AT1 marker) (tdTomato, red; PDPN, cyan; DAPI, blue). Scale bars: 200 μm. (**D**) qPCR analysis of *Mki67* from mouse AT2 organoids. (**E**) qPCR analysis of *Pdpn* from mouse AT2 organoids. One-way ANOVA with Tukey’s post hoc analysis was used in **D** and **E**. Data are presented as mean ± SD (*P < 0.05).

To further assess the functional contributions of α7nAChR-driven WNT7B to alveolar regeneration, we also observed changes in proliferative and differentiative capacity in lineage-labeled AT2 cells (Figure 7A). As expected, immunofluorescence analysis of Ki67^+^tdTomato^+^ cells (proliferative AT2 cells, Figure 7B) and PDPN^+^tdTomato^+^ cells (AT1 cells, Figure 7C) revealed a reduction in the proliferative capacity of AT2 cells on day 10 and differentiation of AT2-to-AT1 cells on day 12 in alveolar organoids after knockout of α7nAChR on AT2 cells, but adding WNT7B partly restored this capacity. We also confirmed that expression of both *Mki67* and *Pdpn* at mRNA level in WNT7B-treated *Sftpc-cre^ERT2^Chrna7^fl/fl^R26R^tdTomato^* organoids was increased compared with that in PBS-treated *Sftpc-cre^ERT2^Chrna7^fl/fl^R26R^tdTomato^* organoids (Figure 7, D and E). Taken together, these results strongly support that α7nACh is requisite for AT2 cell proliferation and subsequent differentiation to AT1 cells after lung injury and that WNT7B mediates this effect.

## Discussion

Reducing inflammatory damage and improving alveolar epithelium regeneration are two key points to promoting lung repair in ARDS/ALI (1). We have found that many nicotinic acetylcholine receptors were expressed on AT2 and AT1 cells during homeostasis and injury in mice (data not shown). Of them, α7nAChR has been reported to attenuate pulmonary inflammation and alveolar damage in ALI induced by various insults (7–10). In addition to the antiinflammatory function of α7nAChR, evidence supports that α7nAChR plays important role in lung development (18, 20, 21). However, little is known about the physiological function of α7nAChR in alveolar regeneration, which relies on the proliferation and proliferation of AT2 cells (22, 23). Here, we focused on the role of α7nAChR in the alveolar epithelium regeneration. We found that α7nAChR expression of AT2 cells was upregulated in response to LPS-induced lung injury. Furthermore, our data demonstrated that knockout of α7nAChR on AT2 cells impeded the proliferation and subsequent differentiation of AT2 cells via a canonical WNT7B signaling during the alveolar regeneration process, accompanied by aggravated lung inflammation and hampered recovery of lung tissue structure. Our findings provide insights into how α7nAChR controls pulmonary inflammation and promotes alveolar regeneration, eventually facilitating the repair of lung structure and function. In addition, we have measured the markers of fibrosis in the animals and did not observe induced expression of TGF-β or α-SMA (Supplemental Figure 10). Thus, it could be possible to utilize vagus stimulation or α7nAChR agonist to alleviate ALI without inducing fibrosis.

Alveolar epithelium repair largely depends on the responsive and effective proliferation of AT2 cells, followed by effective differentiation into AT1 cells (22, 23). We confirmed the presence of α7nAChR in AT2 cells as reported previously (25). Intriguingly, α7nAChR expression in AT2 cells was significantly upregulated in response to LPS-induced lung injury but not LPS-induced sepsis (data not shown), indicating that α7nAChR might be a key regulator of properties of the AT2 cells in ALI (Figure 1). Similarly, a previous study (26) has demonstrated an increase in α7 transcripts in CD45^–^ lung interstitial cells 3 days after LPS challenge. In addition, knockout of α7nAChR on AT2 cells kept a high inflammatory status in the lung and impeded the recovery of lung pathological injury after LPS assault (Figure 2), suggesting that α7nAChR participates in the lung reparative process.

To investigate how α7nAChR controls the cellular fate of AT2 cells after injury, we further evaluated the proliferative and differentiative capacity of AT2 cells after LPS insult. Our previous findings (29) have demonstrated that vagal innervation in lung facilitated AT2 cell expansion via α7nAChR. Likewise, we discovered that knockout of α7nAChR on AT2 cells reduced the percentage of AT2 cells and the number of proliferating AT2 cells in the mouse lung treated with LPS (Figure 3, A–H), but not AT1 cells or club cells (Supplemental Figure 1, B–E). However, it should be pointed out that AT2 cells numbers were directly counted in multiple sections of each animal but not by stereology, which could be modified in our future studies. Of note, the number of alveolar organoids and proliferating AT2 cells was increased by a selective α7nAChR agonist but remarkably decreased by an α7nAChR antagonist (Figure 4 and Supplemental Figure 5). It is worth noting that a AT1 cell marker was significantly elevated by activation of α7nAChR in the lineage-labeled AT2 cells in an in vitro transdifferentiation assay and that this effect was blocked by an α7nAChR antagonist (Supplemental Figure 2A). The disparity between the in vivo and in vitro findings could be ascribed to the fragility of AT1 cells during lung tissue dissociation after injury and the time frame and methods chosen for observing differentiation. As a matter of fact, we found a significant increase in the number of lineage-labeled AT1 cells after selective α7nAChR agonist treatment on day 14 after injury (Figure 3, I and J, and Supplemental Figure 3).

Overall, our data suggest that α7nAChR expressed on AT2 cells is critical for the renewal of alveolar regeneration through promotion of AT2 cell proliferation and subsequent differentiation. Consistent with our findings, many authors have noticed that α7nAChR is closely associated with an enhanced proliferative property of various cells, including normal cells, cancerous cells (13, 17), and stem cells (30–32). On the contrary, activation of α7nAChR results in a reduction of proliferation in adipose-derived stem cells (12), inhibited osteogenic differentiation in human periodontal ligament cells (33), chondrogenic differentiation in bone mesenchymal stem cells (34), and inhibited proliferation and differentiation in osteoblasts (35). Inconsistently, it has been reported that selective activation of α7nAChR causes a limited proliferation but promotes the differentiation of basal epithelial cells in airway regeneration (21) and of neural stem cells after stroke (36). Thus, the effect of α7nAChR activation on the cellular fates depends on cells identities.

Importantly, we screened out WNT7B signaling as a vital pathway dedicated to α7nAChR-promoted alveolar regeneration. Our data revealed that knockout of α7nAChR in AT2 cells downregulated WNT7B expression (Figure 5, A and B); however, vagus helped to retain higher activation of a canonical WNT7B signaling by activating α7nAChR in AT2 cells (Figure 5, C–H, and Supplemental Figure 9), leading to the conclusion that vagal α7nAChR maintains a canonical WNT7B signaling in AT2 cells. Similarly, previous studies have revealed that α7nAChR agonists could regulate the proliferation and differentiation of osteoblasts (35) and periodontal ligament cells (33, 37) by regulating the WNT/β-catenin signaling pathway or exerts its neuroprotective function in Parkinson’s disease model via WNT/β-catenin signaling (38). In turn, there were data to suggest that the promoter region of *Chrna7* contains binding motifs that are typical targets for the WNT/β-catenin signaling pathway (39) and inhibition of β-catenin expression lowered the level of α7nAChR protein (40). WNT induces presynaptic colocalization of α7nAChR in hippocampal neurons (41) and controls the translocation of ACR16/α7nAChR to synapses (42). These data reveal a crosstalk between a7nAChR and the WNT/β-catenin signaling pathway. Furthermore, α7nAChR is a homomeric calcium channel. The WNT/β-catenin signaling pathway can be regulated by calcium/calmodulin-dependent protein kinase II (CaMK II) (43). In addition, activation of calcium channel leads to calcium influx, which activates CaMK II in cascade. Thus, we proposed that α7nAChR possibly regulated WNT7B signaling by promoting calcium influx and CaMK II activation.

Intriguingly, in our study, loss of α7nAChR in AT2 cells blocked the AT2 lineage–labeled alveolar organoid formation (Figure 6) by impeding the proliferative and subsequent differentiative process of lineage-labeled AT2 cells (Figure 7), whereas activation of canonical WNT7B signaling promoted it, suggesting that autocrine WNT7B signaling is integral for α7nAChR-promoted alveolar regeneration. Likewise, WNT7B can enhance both self-renewal and osteogenic differentiation of bone mesenchymal stem cells (44). Given that WNT7B signaling was regulated by vagal α7nAChR, we further concluded that α7nAChR-driven WNT7B signaling is integral for α7nAChR-promoted alveolar regeneration. Taken together, WNT7B signaling plays an essential role in α7nAChR-mediated alveolar regeneration by helping AT2 cells to proliferate and differentiate sequentially, ultimately restoring the alveolar structure. There are multiple lines of evidence (24, 45, 46) that collectively demonstrate an obvious effect of WNT7B in lung development and the reparative process after injury.

Given the result showing that other growth factors expressed in mesenchymal cells, such as *Fgf10*, were significantly downregulated in the lungs of *Sftpc^cre^Chrna7^fl/fl^* mice (Figure 5A), and our previous study showing that vagal α7nAChR mediated expression of *Fgf10* (29), we propose that activation of α7nAChR not only activated autocrine canonical WNT7B signaling cascades, but may also signal to neighboring cells to change the stem cell niche (Figure 5A), which may, in turn, support AT2 cell self-renewal. It is highly likely that α7nAChR promotes AT2 cells to proliferate not only by its direct effect on AT2 cells, but also by modulating stem cell niche that, in turn, supports AT2 self-renewal. Taken together, the findings in this study extend our understanding of the role of α7nAChR in the regulation of AT2 cell proliferation and differentiation, which promotes alveolar regeneration through WNT7b signaling.

## Methods

Reagents and materials are listed in Supplemental Table 1.

### Animals.

α7nAChR-knockout (*Chrna7*^–/–^) mice were purchased from The Jackson Laboratory (B6.129S7-*Chrna7*^tm1Bay^/J). *Sftpc^cre^* mice were provided as a gift from Kaifeng Xu (Chinese Academy of Medical Sciences, Beijing, China) (47). *Sftpc-cre^ERT2^* (B6.129S-*Sftpc*^tm1(cre/ERT2)Blh^/J) mice were a gift from Shaoxi Cai (Chronic Airways Diseases Laboratory, Department of Respiratory and Critical Care Medicine, Nanfang Hospital, Southern Medical University, Guangzhou, China). Ai9 (B6.Cg-*Gt(ROSA)26Sor*^tm9(CAG-tdTomato)Hze^/J) mice were a gift from Zilong Qiu (Center for Excellence in Brain Science and Intelligence Technology, Institute of Neuroscience, State Key Laboratory of Neuroscience, Chinese Academy of Sciences). *Chrna7^fl/fl^* mice were generated by the Transgenic Mouse Facility led by Yan Zhang in the Institut Pasteur of Shanghai. *Sftpc^cre^Chrna7^fl/fl^* mice, *Sftpc-cre^ERT2^R26R^tdTomato^* mice, and *Sftpc-cre^ERT2^* *Chrna7^fl/fl^; R26R^tdTomato^* mice were generated in-house. Female and male mice aged 6–10 weeks were used in the experiments.

### Tamoxifen administration.

Tamoxifen was dissolved in corn oil with a 20 mg/mL stock solution. Tamoxifen was given via intraperitoneal injection, with a dosage of 0.1 mg/g body weight. The numbers and dates of tamoxifen administration are indicated in Figures 1 and 5.

### ALI model and intervention.

The 6- to 10-week-old mice were housed for 12-hour dark/light cycles with free access to food and water before intervention. Then, mice were anesthetized by intraperitoneal injection of Avertin (Sigma-Aldrich). Subsequently, LPS (2.5 mg/kg), GTS-21 (4 mg/kg), or PBS with equal volume was intratracheally or intranasally delivered at time points indicated in Figures 1, 4, 5, and 6. At indicated time points, mice were humanely sacrificed using Avertin.

### Histology and immunofluorescence analysis of lung tissue.

Mouse lung tissues were routinely perfused, inflated, and fixed with 4% paraformaldehyde. Cryosections and paraffin sections were used for histology and immunofluorescence analysis. Sectioned lung tissues were stained with H&E, immunofluorescence stained, or immunohistochemically stained. After antigen retrieval, sections were blocked with 5% BSA in 0.2% Triton-X100/PBS at room temperature for 1 hour. Primary antibodies were incubated overnight at 4°C at the indicated dilutions: rabbit anti–pro-Sftpc, goat anti-Sftpc, eFluor570-rat anti-Ki67, AF647 anti-mouse PDPN, mouse monoclonal antibody against mCherry, and rabbit anti-RFP. Corresponding fluorescence-coupled secondary antibodies were incubated, followed by DAPI staining. Fluorescence images were acquired using a confocal microscope (Olympus IXplore SpinSR and Olympus FV3000). Images were processed with Fiji software. The method of cell counting analysis for lung tissue was from Choi et al. (48).

### Lung injury score.

The lung pathological injury was graded independently by two researchers in a blinded manner as described previously (29).

### Preparation of lung single-cell suspension.

Lung tissues were dissociated following a previously published protocol with modifications (29). Briefly, mice were humanely sacrificed and perfused with cold PBS through their right ventricles to remove blood cells in the lung. Subsequently, 1 mL dispase II (15 mg/mL) was instilled into the lung via trachea until the lung inflated. The trachea was carefully removed, and then the lungs were minced and incubated in a 37°C shaking incubator for 45 minutes in 2 mL of 2 μg/mL collagenase/dispase II containing 1% DNase I. The single-cell suspension was obtained after passing cells through a 100 μm cell strainer and then a 40 μm cell strainer, after which cells were centrifuged at 142 g for 5 minutes. The cell pellet was resuspended in 2 mL RBC lysis buffer and incubated at room temperature for 2 minutes to remove red blood cells. 6 mL DMEM/F12 was added, and 500 μL of FBS was slowly added in the bottom of tubes. Cells were centrifuged at 319 g for 5 minutes at 4°C and was resuspended in DPBS with 10% FBS for further staining.

### Flow cytometry and cell sorting for AT2 and lung mesenchymal cells.

After preincubating for 15 minutes with anti-mouse CD16/32 antibodies (eBioscience), Fixable Viability Stain 780 (BD Bioscience) was used to exclude dead cells. Subsequently, lung cells were labeled with antibodies. For staining indirectly labeled antibodies, the primary antibodies were stained for 30 minutes and washed out followed by the secondary antibodies staining. The antibodies used were as follows: APC/Fire 750 anti-mouse F4/80, BV650 anti-mouse/human CD11b, BV421 anti-mouse Ly6G, PE/Cyanine7 anti-mouse Ly6C, BV421 anti-mouse Ki67, PE anti-mouse PDPN, APC anti-mouse CD31, APC anti-mouse CD45, FITC anti-mouse CD326/EPCAM, BV510 donkey anti-rabbit IgG, AF488 donkey anti-rabbit IgG, rabbit anti-pro-Sftpc (Millipore), rabbit anti-mouse CCSP (Proteintech), CF633-α-Bungarotoxin (Biotium) (all others from Biolegend). Debris and aggregates were≠ excluded and live cells were detected on a LSRFortessa (BD Biosciences) and then analyzed by FlowJo X 10.0.7 software (Tree Star Inc.). BD FACS Aria II appliances were used for AT2 lineage–labeled cell sorting; the sorting strategies are shown in the figures. For isolation of lung mesenchymal cells, cells negatively isolated by a CD31 via MagCellect Magnet column (R&D Systems) were further negatively sorted by EPCAM and CD45 via a MagCellect Magnet column.

### RNA-Seq and analysis.

After being sorted from indicated groups, the Sftpc lineage–labeled cells were harvested, and the total RNA was extracted using a RNeasy Micro Kit (Qiagen) according to the manufacturer’s instructions. After quality testing, RNA-Seq was performed on an MGISEQ-2000 platform. Differentially expressed genes were analyzed with GraphPad. The protein interaction network and pathway analysis were performed in STRING (https://cn.string-db.org/).

### Primary alveolar organoid culture.

Lung organoids were established based on a previous report with modifications (48). Briefly, freshly sorted lineage-labeled cells (CD45^–^CD31^–^EPCAM^+^tdTomato^+^) were resuspended in basic media (advanced DMEM/F12 supplemented with 10% FBS and mixed with lung mesenchymal cells (CD45^–^CD31^–^EPCAM^–^) at a ratio of 1:6, followed by resuspension in growth factor–reduced Matrigel (BD Biosciences) at a ratio of 1:5. A 10 μL mixture was placed in a prewarmed 24-well Transwell insert with a 0.4 mm pore (Corning) or 48-well cell culture plate at 37°C for 20 minutes. Approximately 2 × 10^3^ to 5 × 10^3^ Sftpc^+^ cells were seeded in each insert. 500 μL or 250 μL organoid media (listed in Supplemental Table 2) was placed in the lower chamber, and medium was changed every 3 days with or without LPS (1 μg/mL, Sigma-Aldrich), GTS-21 (10 μmol/L, Abcam), MLA (10 μmol/L, Abcam), WNT7B (100 ng/mL, referred to in refs. 49, 50; Novus Biologicals). R-Spondin-1 (500 ng/mL, Peprotech) and ROCK inhibitor Y-27632 (5 μmol/L, APExBio) were added in the medium for the first 3 days of culture. Colony-forming efficiency and size of organoids were analyzed at day 10 after plating if there is no specific description.

### Immunofluorescence staining for organoids.

To perform the immunostaining of organoids, organoids were cultured on a prewarmed 24-well Transwell insert and stained as previously reported with modifications (51). Organoids were fixed for 2 hours in 4% paraformaldehyde, permeabilized, and blocked for 2 hours in 0.5% Triton X-100 plus 1% BSA. Subsequently, organoids were incubated with primary antibodies overnight at 4°C. Then, organoids were incubated with secondary antibodies overnight at 4°C. After immunostaining, the Transwell inserts were mounted on the glass slide with Fluoroshield (Sigma-Aldrich). Samples were imaged on a confocal microscope (Olypmus IXplore SpinSR). Images were processed with Fiji software.

### EdU incorporation assays in organoids.

Lineage-labeled AT2 cells from *Sftpc-cre^ERT2^R26R^tdTomato^* mice intraperitoneally injected by tamoxifen were isolated at day 4 after final injection. Organoids were treated with EdU (10 μmol/L) at indicated time for 3 hours. EdU staining was performed according to the manufacturer’s instructions (Beyotime).

### Primary AT2 cell culture on 2D plates.

Primary mouse AT2 cells were isolated and sorted as described above. Sorted AT2 cells were directly cultured on fibronectin-coated 24-well plates for 6 hours before treatment according to a previous study, with modifications (51). The DMEM/F12 medium containing 10% FBS was used for culturing AT2 cells. On days 1, 3, and 5, cells were lysed, and total RNA was extracted and processed for qPCR.

### ELISA.

WNT7B protein was released from homogenized mouse lung to supernatant. The supernatant was diluted and measured using a mouse WIN7B ELISA kit (Cusabio) according to the manufacturer’s instructions.

### Complete blood count.

Using a multispecies hematology instrument (Mindray BC-5300vet), we detected the percentage of neutrophils, lymphocytes, monocytes, and eosinophils in the blood.

### qPCR.

Total RNA of homogenized lungs was extracted using TRIzol reagent (Invitrogen). Total RNA of sorted AT2 cells or organoids was isolated using a RNeasy Micro Kit (Qiagen) according to the manufacturer’s instructions and then converted into cDNA using the FastKing RT Kit (Tiangen). Then, qPCR analysis was carried out using SYBR Green master mix (Yeasen) in a Applied Biosystems RT-PCR 384 System. The primers are listed in Supplemental Table 3.

### Statistics.

Statistical analyses were performed using Prism software package version 8.0 (GraphPad). Statistical significance was calculated using 2-sided, 2-tailed *t* test or 1-way or 2-way ANOVA. Data shown are representative of at least 3 independent experiments and are shown as the mean ± SD. *P* values of less than 0.05 were regarded as significant.

### Study approval.

The protocols were approved by the Committees on Animal Research of Zhongshan Hospital and the Institut Pasteur of Shanghai.

### Data availability.

Values for all data points in graphs and RNA-Seq data are reported in the Supporting Data Values file.

## Author contributions

XC, YS, and XS designed the experiments, interpreted the data, and wrote the manuscript. XC, CZ, and TW performed most of the experiments and data analysis. XC and CZ bred the mice. XC, CZ, and TW shared the co–first authorship; order of co–first authors was determined based on their contribution to the study. JC, TP, and ML helped to develop, breed, and identify gene editing mice. LW and JS helped to create the organoids model. CC offered valuable scientific discussions and technical support. YZ provided the *Chrna7^fl/fl^* mouse line. YS and XS supervised the study, provided scientific insight, and reviewed and edited the manuscript.

## Supplementary Material

Supplemental data

Supporting data values

## Version 1 07/06/2023

In-Press Preview

## Version 2 08/08/2023

Electronic publication
