# Supplementary material for: α7nAChR activation in AT2 cells promotes alveolar regeneration through WNT7B signaling in acute lung injury
Source: JCI Insight. 2023 Aug 8;8(15):e162547. doi: 10.1172/jci.insight.162547 (PMC10445688; doi:10.1172/jci.insight.162547)
Supplement: Supplemental data [file jciinsight-8-162547-s087.pdf]

Supplemental Figures

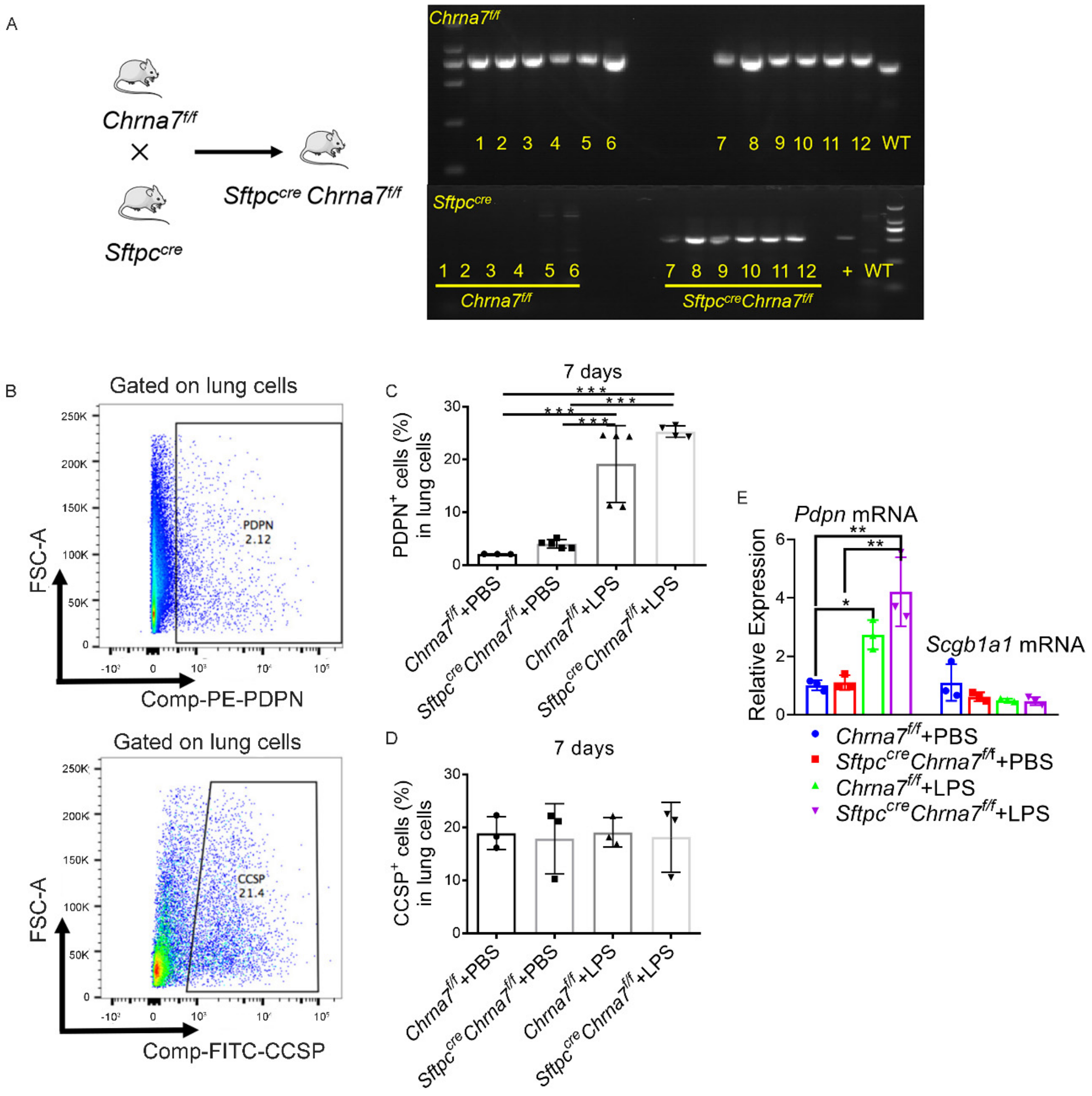

Supplemental Figure 1. A. *Sftpc*^{cre} mice were mated with *Chrna7*$^{f/f}$ mice to specifically knockout α7nAChR in AT2 cells and their offspring were identified by PCR; B. PBS or LPS (2.5mg/kg) was intratracheally delivered to *Sftpc*^{cre}*Chrna7*$^{f/f}$ mice or *Chrna7*$^{f/f}$ mice and was followed up for 7 days. The flow cytometry was used to detect PDPN^{+} cells (AT1 marker) and CCSP^{+} cells (Club cells); C. The statistical results of the percentage of PDPN^{+} cells in mouse lung; D. The statistical results of the percentage of CCSP^{+} cells in mouse lung; E. The relative gene expression of *Pdpn* and

Scgb1a1 in lung tissue homogenate was tested by qPCR. 1-way ANOVA with Tukey's post hoc analysis was used in C-E. Data are representative of at least three independent experiments and are presented as mean ± SD. (N = 3-5; $^{*}P < 0.05$, $^{**}P < 0.01$, $^{***}P < 0.001$).

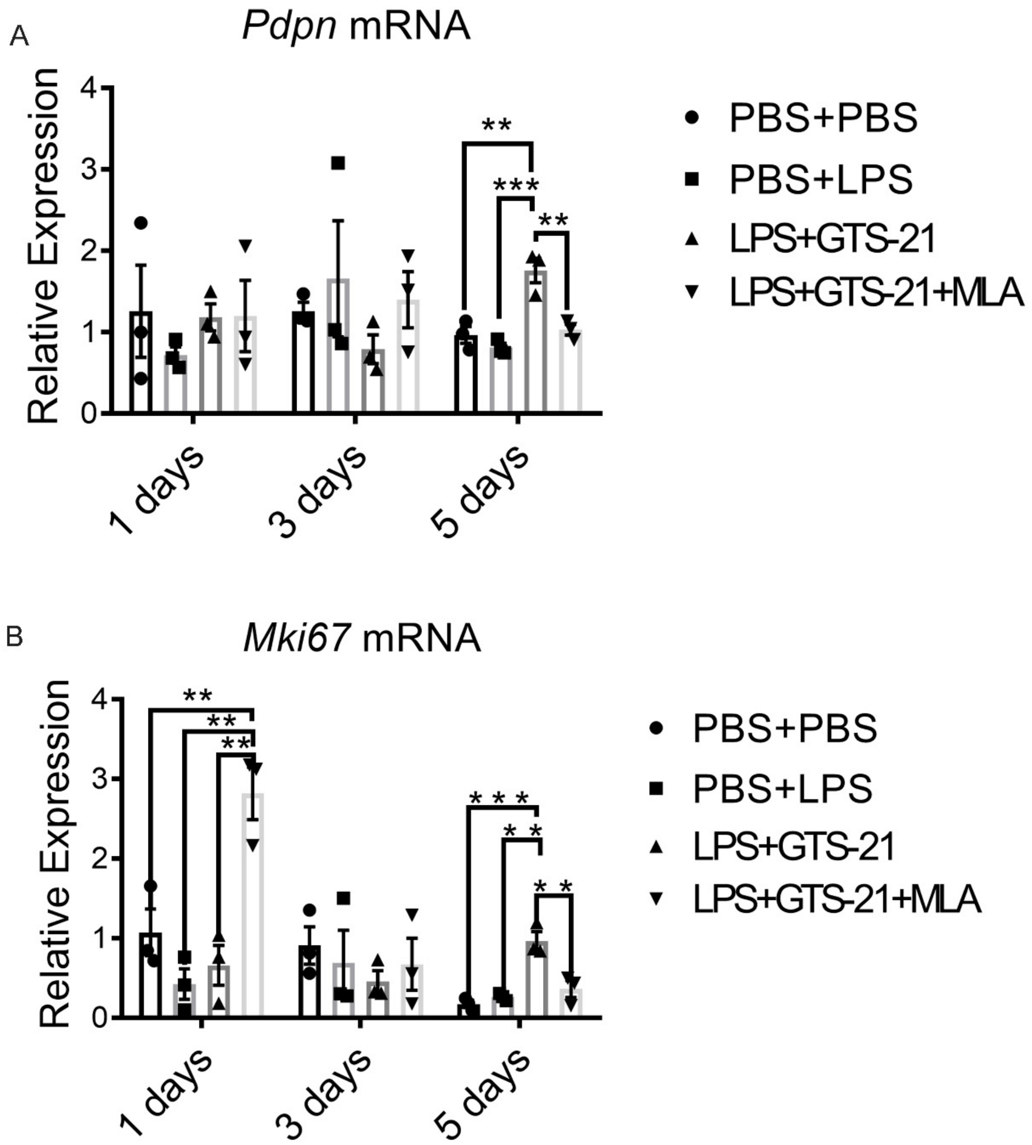

Supplemental Figure 2. Primary mouse AT2 cells were purified by fluorescence activated cell sorter (FACS) and then treated with PBS, LPS (1 μg/mL), GTS-21 (10 μmol/L), or MLA (10 μmol/L) in vitro and the gene expression of *Pdpn* (A) and *Mki67* (B) was quantified by qPCR at indicated time points. 1-way ANOVA with Tukey's post hoc analysis was used. Data are

representative of at least three independent experiments and are presented as mean ± SD. ($^{**}P$ < 0.0 1***, P < 0.001).

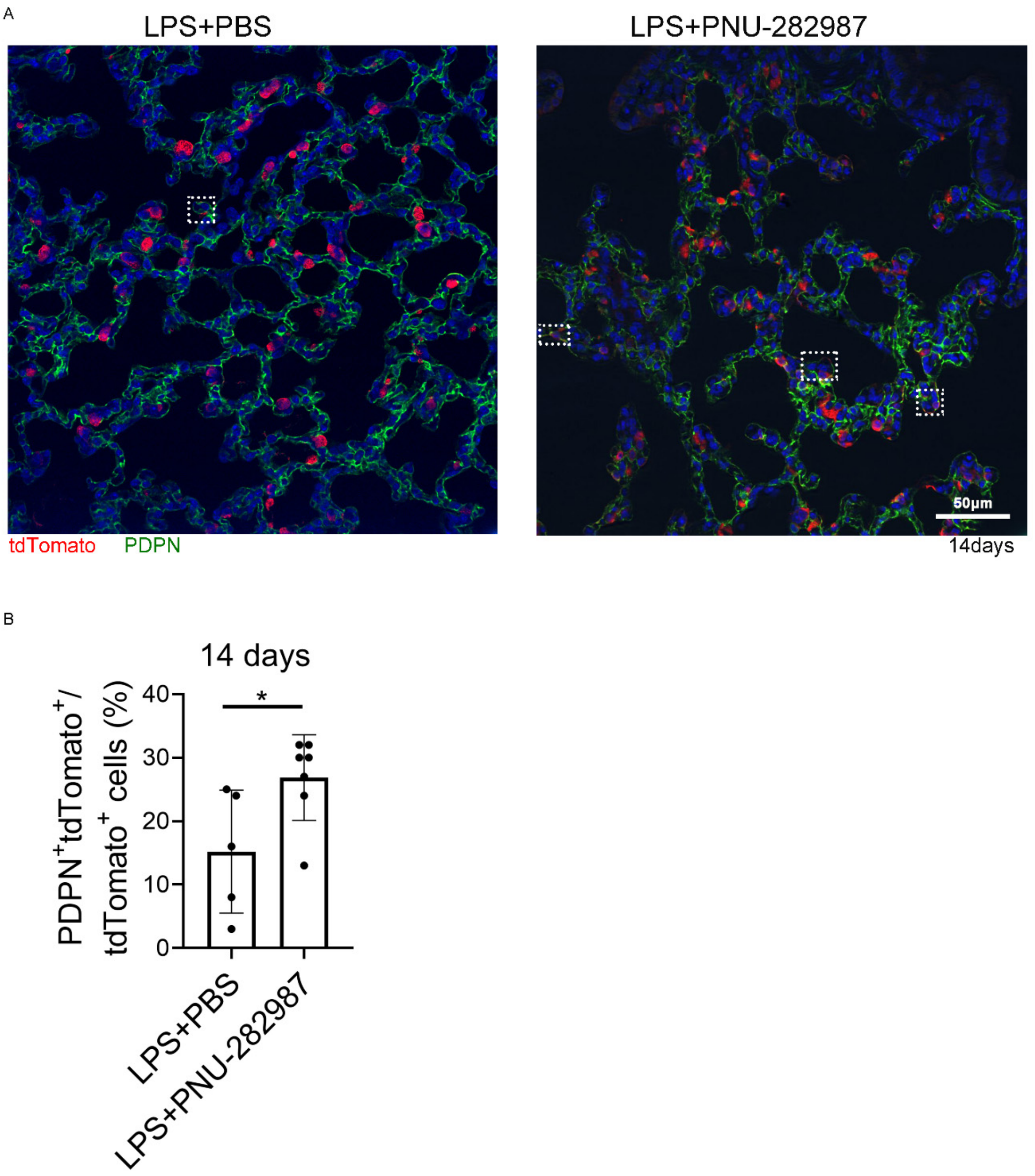

Supplemental Figure 3. A. Representative IF images showing podoplanin$^+$ (PDPN$^+$) AT1 cells differentiation from *Sftpc* lineage-labeled cells on day 14 post injury in the lung of indicated groups: tdTomato (red), PDPN (green), and DAPI (blue). Scale bars, 50 μm; B. Quantification of lineage-labeled PDPN$^+$ AT1 cells in (A). Each individual dot represents one section. 2-sided *t* test

was used. Data are representative of at least three independent experiments and are presented as mean ± SD. (N = 5-7; $^*P < 0.05$).

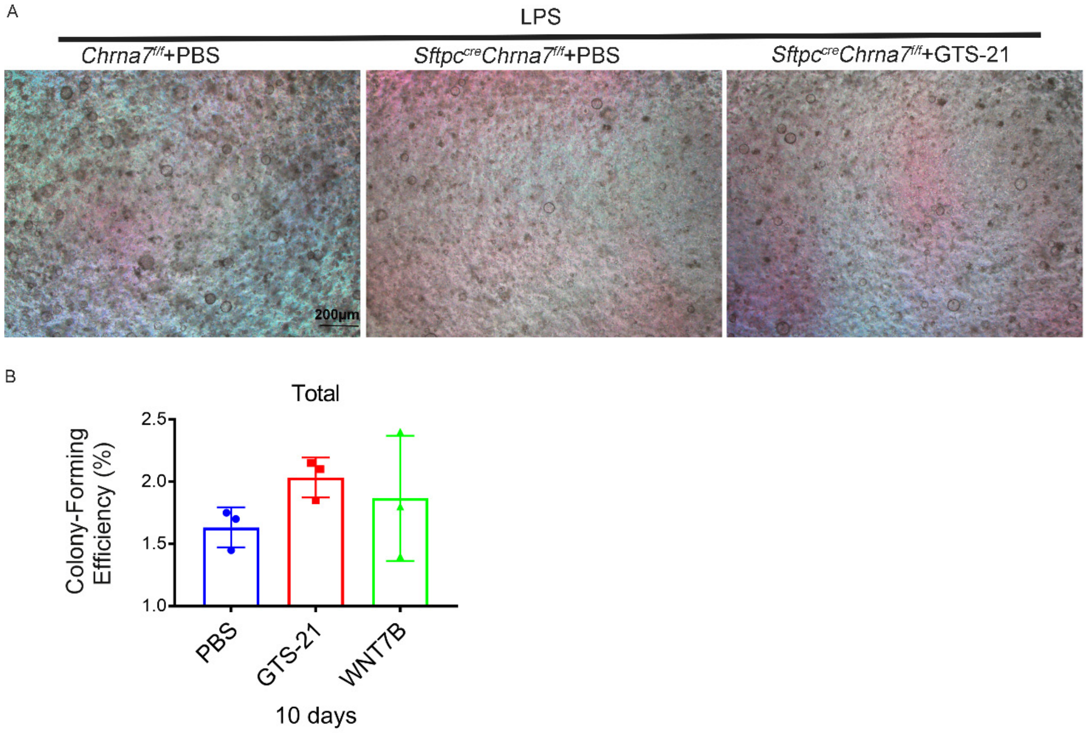

Supplemental Figure 4. Representative bright-field images of alveolar organoids. A. We co-cultured lung mesenchymal cells ($CD45^-CD31^-EPCAM^-$) with AT2 cells ($CD45^-CD31^-EPCAM^+$) isolated from *Sftpc*^{cre}*Chrna7*$^{f/f}$ mice or Chrna7$^{f/f}$ mice to create 3D organoids model. LPS (1 μg/mL) and GTS-21 (selective α7nAChR agonist, 10 μmol/L) was added on day 3, then organoids were followed for additional 5 days. Scale bars, 400 μm; Data are representative of at least three independent experiments. B. Organoid co-culture of *Sftpc* lineage-labeled cells ($CD45^-CD31^-EPCAM^+tdTomato^+$) with lung mesenchymal cells ($CD45^-CD31^-EPCAM^-$) isolated from α7nAChR knockout (*Chrna*$^{-/-}$) mice. PBS, GTS-21(10 μmol/L), and WNT7B (100 ng/mL) were added to the indicated group. The total colony formation efficiency of alveolar organoids was analyzed. Each individual dot represents one experiment; 1-way ANOVA analysis was used. Data are presented as mean ± SD.

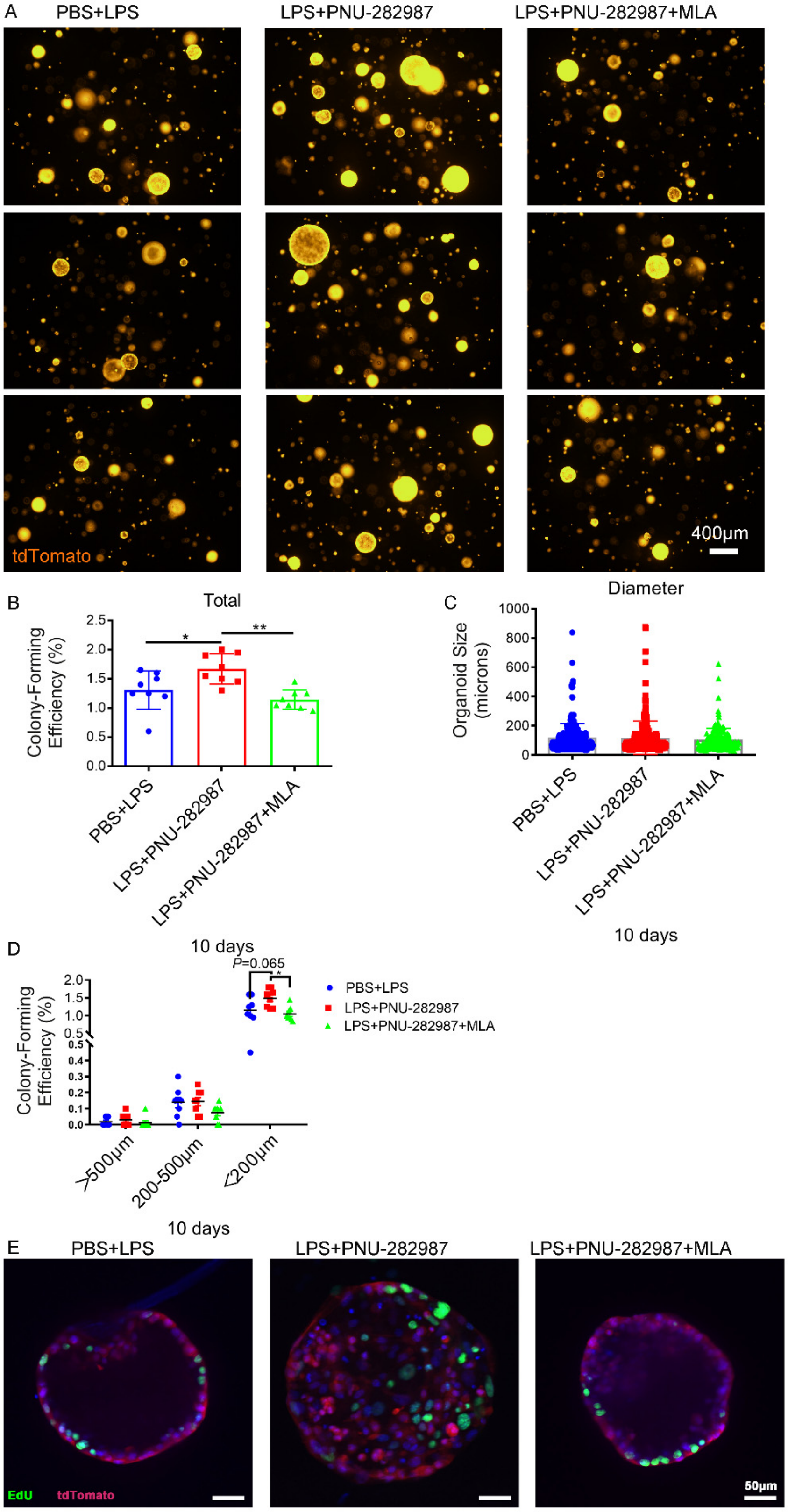
A
PBS+LPS
LPS+PNU-282987
LPS+PNU-282987+MLA
tdTomato
400μm
B
Total
Colony-Forming Efficiency (%)
2.5
2.0
1.5
1.0
0.5
0.0
*
**
PBS+LPS
LPS+PNU-282987
LPS+PNU-282987+MLA
C
Diameter
Organoid Size (microns)
1000
800
600
400
200
0
PBS+LPS
LPS+PNU-282987
LPS+PNU-282987+MLA
10 days
D
10 days
P=0.065
*
PBS+LPS
LPS+PNU-282987
LPS+PNU-282987+MLA
Colony-Forming Efficiency (%)
>500μm
200-500μm
<200μm
10 days
E
PBS+LPS
LPS+PNU-282987
LPS+PNU-282987+MLA
EdU
tdTomato
50μm

Supplemental Figure 5. A. Representative fluorescence images of AT2 organoids captured on day 10. LPS (1 μg/mL) was added to simulate lung injury in vitro and methyllycaconitine citrate (MLA, 10 μmol/L) was treated 15 min before PNU-282987 (10 μmol/L). Scale bars, 400 μm; B. Statistical quantification of the total colony formation efficiency of alveolar organoids. Each individual dot represents one experiment from one mouse; C. Statistical quantification of the size of alveolar organoids. Each individual dot represents one organoid; D. Statistical quantification of total colony formation efficiency of alveolar organoids of different sizes; E. Representative fluorescence images showing proliferating cells in AT2 organoids derived from the lungs of lineage tracing mice. Organoids were treated with 5-ethynyl-2'-deoxyuridine (EdU) at an early time point (day 8) for 3 h in cultures. tdTomato (red), EdU (green), and DAPI (blue). Scale bars, 50 μm. 1-way ANOVA with Tukey's post hoc analysis was used in B-D. Data are representative of at least three independent experiments and are presented as mean ± SD. (*$P < 0.05$, **$P < 0.01$).

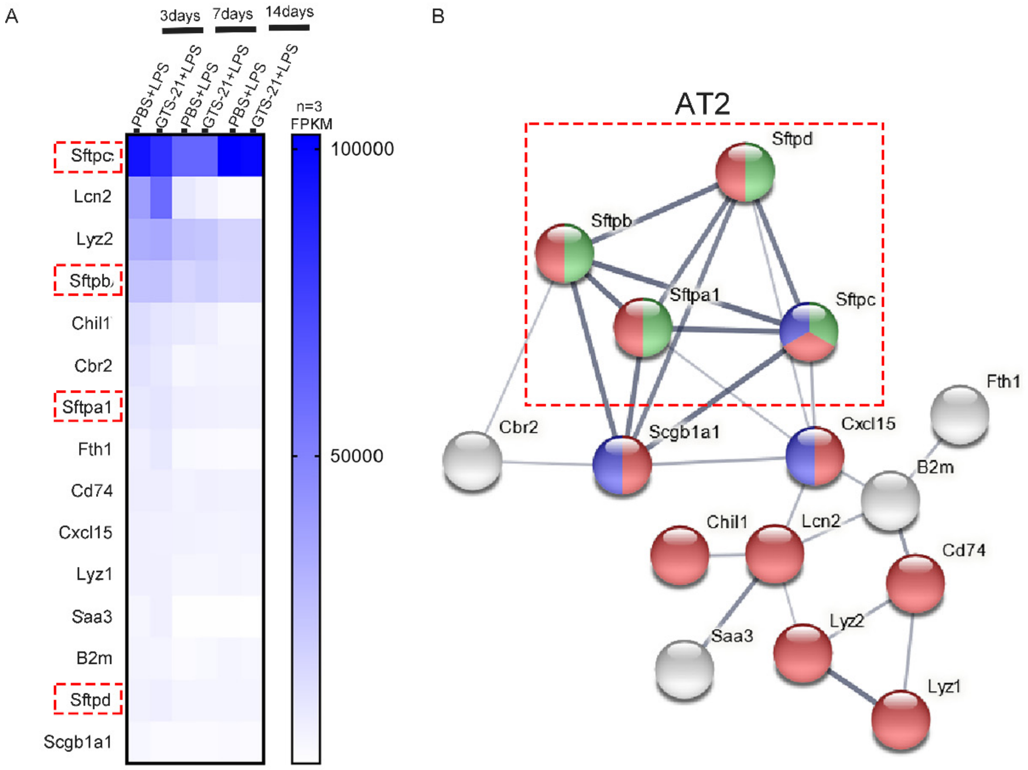

C

Reactome Pathways

pathway	description	count in network	strength	false discovery rate
MMU-5683826	Surfactant metabolism	4 of 21	2.45	2.81e-06

Tissue expression (TISSUES)

tissue	description	count in network	strength	false discovery rate
BTO:0000763	Lung	11 of 1144	1.15	2.38e-08
BTO:0004299	Lung epithelial cell	3 of 6	2.87	1.95e-05

Supplemental Figure 6.RNA-seq analysis of AT2 cells specific genes. Cells were isolated from PBS treated LPS-challenged *Sftpc-cre*^{ERT2}*R26R*^{tdTomato} and GTS-21 treated LPS-challenged *Sftpc-cre*^{ERT2}*R26R*^{tdTomato} mice indicated in Figure 5C. A. Hotmap analysis of the top 15 genes of highly expressed genes in AT2 cells on day 3, day 7, and day 14; B. Protein interaction network among the above 15 genes; C. Reactome pathway and Tissue expression analysis by STRING in the top 15 genes. *N* = 3 in each group.

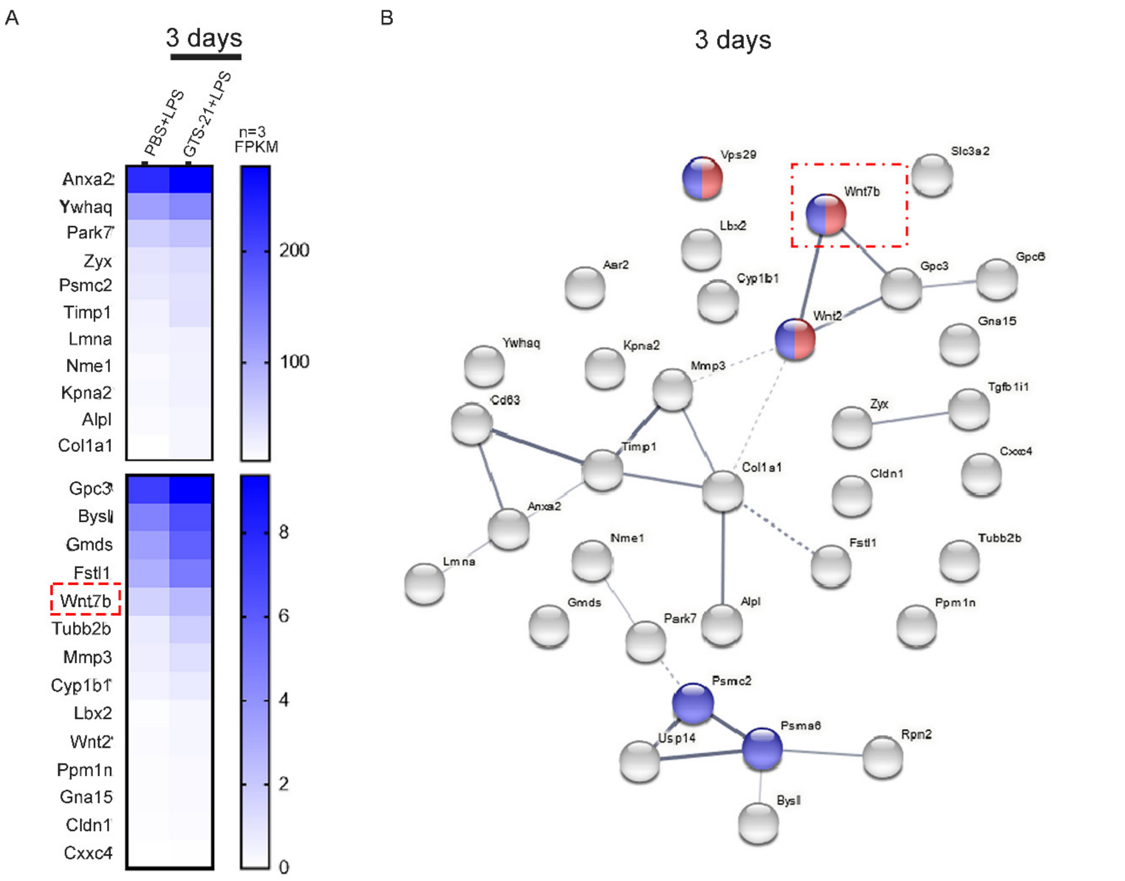

C

Reactome Pathways					
pathway	description	count in network	strength	false discovery rate	
MMU-3238698	WNT ligand biogenesis and trafficking	3 of 25	1.89	0.0172	
MMU-195721	Signaling by WNT	5 of 230	1.15	0.0172	

Supplemental Figure 7.RNA-seq analysis of the differentially upregulated genes of AT2 cells isolated from PBS treated LPS-challenged *Sftpc-cre*^{ERT2}*R26R*^{tdTomato} and GTS-21 treated LPS-challenged *Sftpc-cre*^{ERT2}*R26R*^{tdTomato} mice on day 3 indicated in Figure 5C. A. Hotmap analysis of the differentially upregulated genes of AT2 cells on day 3; B. Protein interaction network among the differentially upregulated genes of AT2 cells on day 3; C. Reactome pathway analysis by STRING in the differentially upregulated genes in AT2 cells on day 3. *N* = 3 in each group.

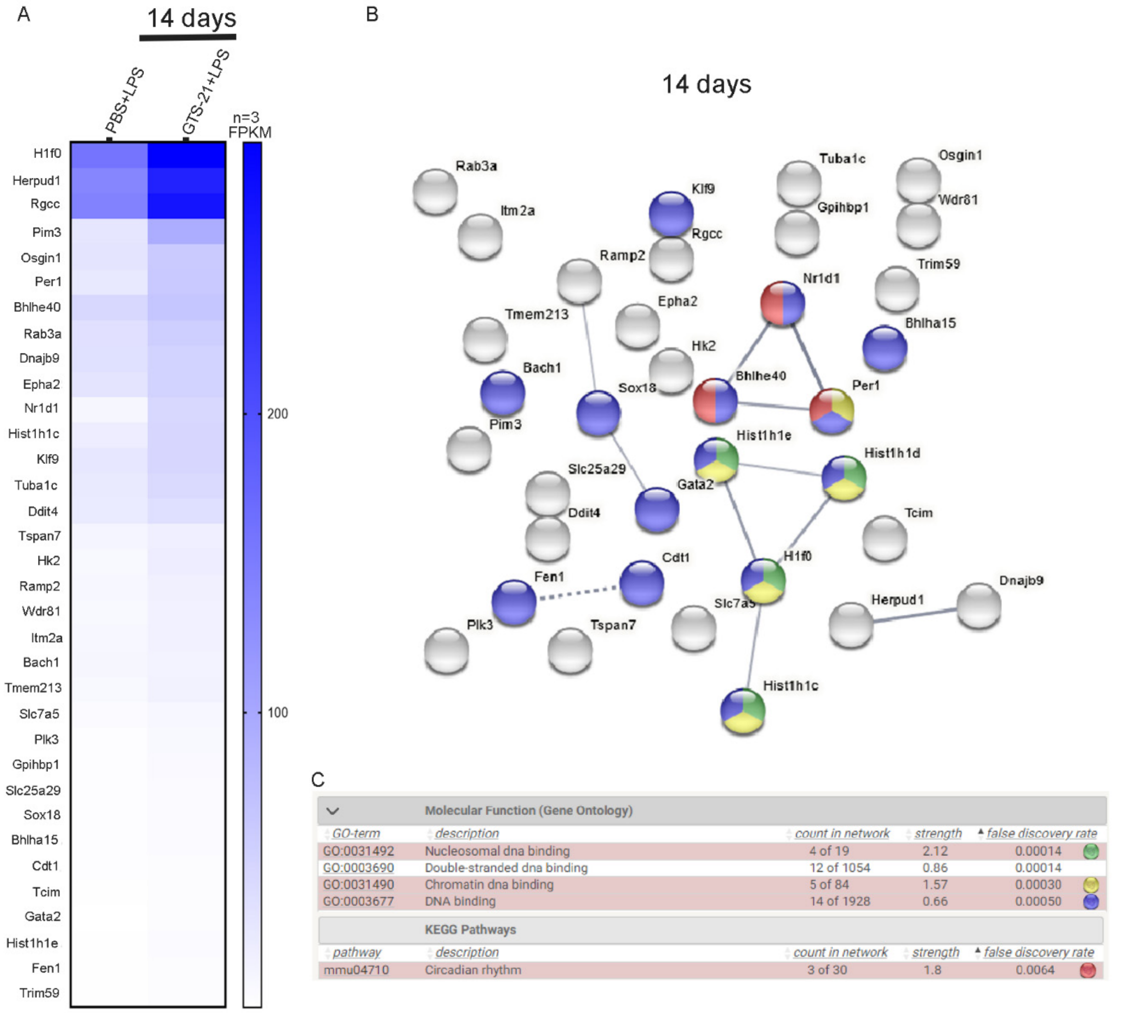

GO-term	description	count in network	strength	false discovery rate
GO:0031492	Nucleosomal dna binding	4 of 19	2.12	0.00014
GO:0003690	Double-stranded dna binding	12 of 1054	0.86	0.00014
GO:0031490	Chromatin dna binding	5 of 84	1.57	0.00030
GO:0003677	DNA binding	14 of 1928	0.66	0.00050

pathway	description	count in network	strength	false discovery rate
mmu04710	Circadian rhythm	3 of 30	1.8	0.0064

Supplemental Figure 8. RNA-seq analysis of the differentially upregulated genes of AT2 cells isolated from PBS treated LPS-challenged *Sftpc-cre*^{ERT2}*R26R*^{tdTomato} and GTS-21 treated LPS-challenged *Sftpc-cre*^{ERT2}*R26R*^{tdTomato} mice on day 14 indicated in Figure 5C. A. Hotmap analysis of the differentially upregulated genes of AT2 cells on day 14; B. Protein interaction network among the differentially upregulated genes of AT2 cells on day 14; C. KEGG pathway and Gene ontology analysis by STRING in the differentially upregulated genes in AT2 cells on day 14. *N* = 3 in each group.

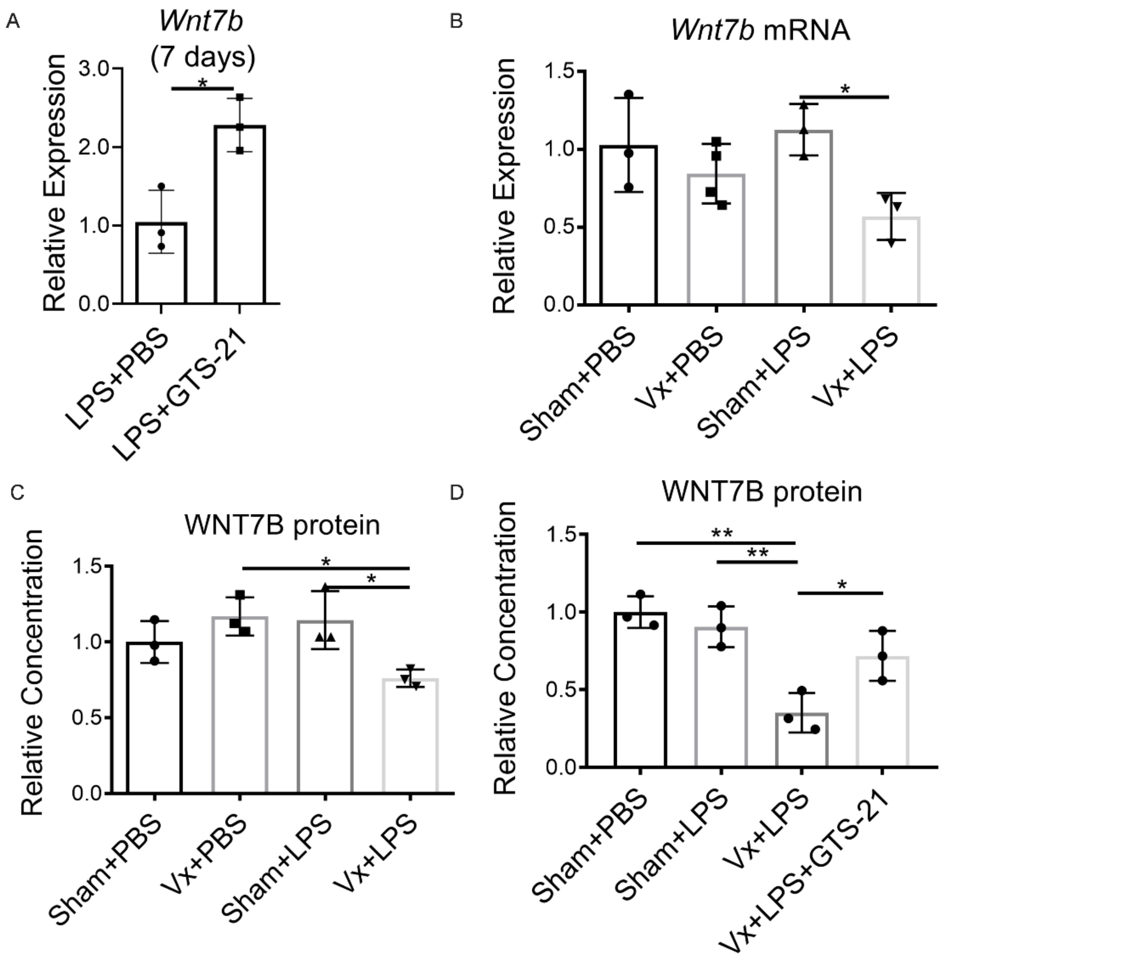

Supplemental Figure 9. Vagal-α7nAChR signaling promotes AT2 to secrete WNT7B. A. qPCR analysis of the *Wnt7b* expression of AT2 cells isolated from PBS treated LPS-challenged *Sftpc-cre*^{ERT2}*R26R*^{tdTomato} and GTS-21 treated LPS-challenged *Sftpc-cre*^{ERT2}*R26R*^{tdTomato} mice on day 7 indicated in Figure 5C (2-sided *t* test was used); B-C. Mice were vagotomized 5 days before LPS insult. PBS or LPS (2.5 mg/kg) was intratracheally delivered to sham or vagotomized (Vx) mice and was followed up for 7 days. The relative gene expression of *Wnt7b* in mouse lung detected by qPCR (B); The relative concentration of WNT7B protein of mouse lung detected by ELISA (C);

D. Mice received a vagotomy or sham operation 5 days before PBS, LPS (2.5 mg/kg), or LPS+GTS-21 (4 mg/kg) challenge. The relative concentration of WNT7B protein in mouse lung was detected by ELISA. 1-way ANOVA with Tukey's post hoc analysis was used in B-D. Data are representative of at least three independent experiments and are presented as mean ± SD. (N = 3-4; $^{*}P < 0.05$, $^{**}P < 0.01$).

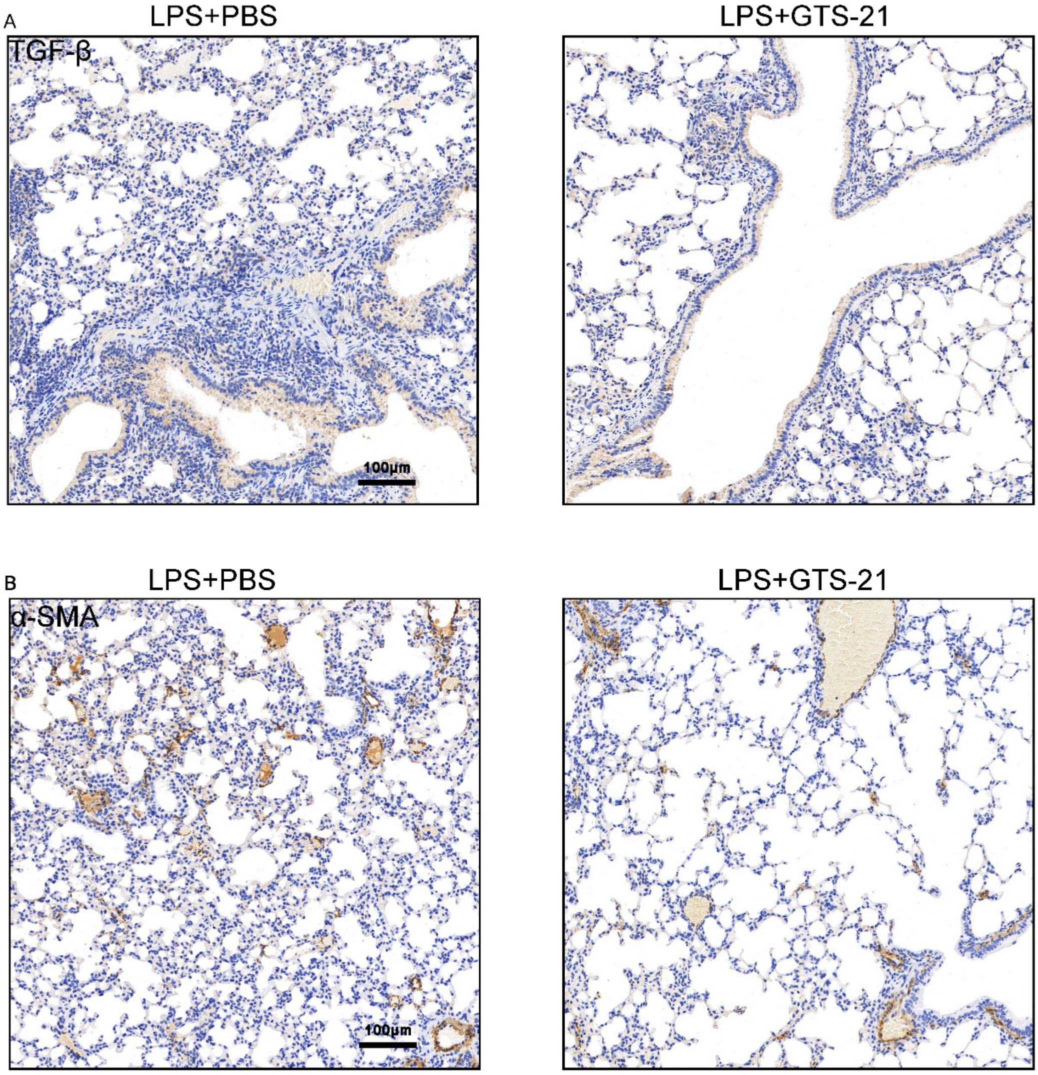

Supplemental Figure 10. IHC analysis of the protein expression of TGF-β (A) and α-SMA (B) in mice lung tissue from PBS treated LPS-challenged *Sftpc-cre*^{ERT2}*R26R*^{tdTomato} and GTS-21 treated

LPS-challenged *Sftpc-cre*^{ERT2}*R26R*^{tdTomato} mice on day 14.

Supplemental Table 1 Reagents and Materials

REAGENT or RESOURCE	SOURCE	IDENTIFIER
**Antibodies**		
rabbit anti-pro-Sftpc	Millipore	AB3786
goat anti-Sftpc	Santa Cruz	sc7706
eFluor™ 570-rat anti-Ki67	eBioscience	41569880
AF647 anti-mouse podoplanin	Biolegend	156203
Rabbit anti-RFP	Rockland	600401379
rabbit anti-mouse CCSP	Proteintech	10490-1AP
AF488 goat anti rabbit	Proteintech	SA00006-2
FITC donkey anti-goat	Servicebio	GB22402
Cy3 donkey anti-rabbit	Servicebio	GB21403
Fixable Viability Stain (FVS) 780	BD Bioscience	565388
anti-mouse CD16/32	eBioscience	14016186
APC/Fire™ 750 anti-mouse F4/80	Biolegend	123151
BV650 anti-mouse/human CD11b	Biolegend	101239
BV421 anti-mouse Ly6G	Biolegend	127627
PE/Cyanine7 anti-mouse Ly6C	Biolegend	128017
BV421 anti-mouse Ki67	Biolegend	652411
PE anti-mouse PDPN	Biolegend	127407
APC anti-mouse CD31	Biolegend	102509
APC anti-mouse CD45	Biolegend	103111

FITC anti-mouse CD326/EPCAM	Biolegend	118207
BV510 donkey anti-rabbit IgG	Biolegend	406419
Alexa Fluor® 488 Donkey anti-rabbit IgG	Biolegend	406416
Biotin anti-mouse CD31 Antibody	Biolegend	102503
Biotin anti-mouse CD45 Antibody	Biolegend	103103
Biotin anti-mouse CD326/EPCAM Antibody	Biolegend	118203
**Chemicals, Peptides, and Recombinant Proteins**		
DispaseII	Sigma-Aldrich	D4693
Collagenase from clostridium histolyticum type 1A	Sigma-Aldrich	C9891
Deoxyribonuclease I from bovine pancreas lyophilized powder	Sigma-Aldrich	DN25
Tamoxifen	Sigma-Aldrich	T5648-1G
Corn oil	Abcone	C67366-250ML
Growth factor-reduced (GFR) Matrigel (10ml)	Corning	356231
Methyllycaconitine citrate (MLA)	Abcam	ab120072
GTS-21 dihydrochloride (DMBX-A)	Abcam	ab120560
Lipopolysaccharides from *Pseudomonas aeruginosa* 10 furified by phenol extraction	Sigma-Aldrich	L9143-10MG
Recombinant human WNT7B	Novus Biologicals	H00007477-P01
CF®633-α-Bungarotoxin	Biotium	Cat#0009
DAPI	Sigma-Aldrich	D9542
MagCellect Streptavidin Ferrofluid	R&D Systems	MAG999B

ACK Lysis Buffer	Beyotime	C3702
**Critical Commercial Assays**		
BeyoClick™ EdU image Kits	Beyotime	C0071S
WNT7B ELISA Kit	Cusabio	CSB-EL026142MO
RNeasy Micro Kit	Qiagen	74004
Transcription Factor Buffer Set	BD bioscience	562574
advanced DMEM/F12	Gibco	12634010
Nicotinamide	sigma	N0636
N-acetylcysteine	sigma	A9165
GlutamMax100×	Invitrogen	12634-034
HEPES	Invitrogen	15630-056
Penicillin / Streptomycin	Invitrogen	15140-122
B-27 supplement	Gibco	1750444
SB202190	APExBio	A1632
Y-27632	APExBio	B1293
A83-01	APExBio	A3133
Noggin	peprotech	120-10C
EGF	peprotech	315-09-500
FGF10	peprotech	100-26-25
FGF7	peprotech	100-19-10
Recombinant Murine R-Spondin-1	peprotech	120-38

**Deposied Data**		
RNA sequencing for *in vivo* AT2-lineage tracing	This paper	deposited in BGI BIG DATABASE
**Experimental Models:Organisms/Strans**		
Mouse/*Sftpc-cre*^{ERT2}	Contact: Prof. Shaoxi Cai	Jackson Laboratory: Stock number:028054
Mouse/*Chrna7*$^{-/-}$		Jackson Laboratory: Stock number:003232
Mouse/*Sftpc*^{cre}	Contact: Prof. Kaifeng Xu	
Mouse/Ai9	Contact: Prof. Zilong Qiu	Jackson Laboratory: Stock number:007909
Mouse/ *Chrna*7$^{f/f}$	Contact: Prof. Yan Zhang	
**Software**		
FlowJo software	Tree Star	
Prism software package version 8.0	GraphPad	

Fiji software		
**Others**		
R&D Systems MagCellectTM Magnet	R&D Systems	MAG997
24-well Transwell insert with a 0.4-mm pore	Corning	3470

Supplemental Table 2 Alveolar Organoid Media Recipe

**Media component**	**Signaling pathway**		**Supplier**	**Catalogue number**
	activation	block		
advanced DMEM/F12	Base medium		Gibco	12634010
Nicotinamide	Co-enzyme precursor		sigma	N0636
N-acetylcysteine	Antioxidant		sigma	A9165
GlutamMax100×	Nutrient		Invitrogen	12634-034
HEPES	Buffer		Invitrogen	15630-056
Penicillin / Streptomycin	Antibiotic		Invitrogen	15140-122
B-27 supplement	a.o. insulin signalling		Gibco	1750444
SB202190		p38-MAPK signalling	APExBio	A1632

Y-27632		ROCK signalling	APExBio	B1293
A83-01		TGF-β signalling	APExBio	A3133
Noggin		TGF-β signalling	peprotech	120-10C
EGF	EGFR signalling		peprotech	315-09-500
FGF10	FGFR2b signalling		peprotech	100-26-25
FGF7	FGFR2b signalling		peprotech	100-19-10
Recombinant Murine R-Spondin-1	Wnt/β-catenin signalling		peprotech	120-38

Supplemental Table 3 Primers Used in the Study

**Primers**	**5' to 3'**
Gapdh(Mus)F	CCCACTAACATCAAATGGGG
Gapdh(Mus)R	CCTTCCACAATGCCAAAGTT
Chrna7(Mus)F	ACAGTACTTCGCCAGCACCA
Chrna7(Mus)R	AAACCATGCACACCAATTCA
Il6(Mus)F	GGCCTTCCCTACTTCACAAG
Il6(Mus)F	ATTTCCACGATTTCCCAGAG
Il1β(Mus)F	CCTGCAGCTGGAGAGTGTGGAT
Il1β(Mus)R	TGCTCTGCTTGTGAGGTGCTG
Tnfα(Mus)F	AAAATTCGAGTGACAAGCCTGTAG
Tnfα(Mus)R	CCCTTGAAGAGAACCTGGGAGTAG
Sftpc(Mus)F	GGAGCACCGGAAACTCAGAA
Sftpc(Mus)R	CTGGCTTATAGGCCGTCAGG
Mki67(Mus)F	ATCATTGACCGCTCCTTTAGGT
Mki67(Mus)R	GCTCGCCTTGATGGTTCCT
Pdpn(Mus)F	ACCGTGCCAGTGTTGTTCTG
Pdpn(Mus)R	AGCACCTGTGGTTGTTATTTTGT
Ccsp/Scgb1a1(Mus)F	ATGAAGATCGCCATCACAATCAC
Ccsp/Scgb1a1(Mus)R	GGATGCCACATAACCAGACTCT
Fgf10(Mus)F	TTTGGTGTCTTCGTTCCCTGT
Fgf10(Mus)R	TAGCTCCGCACATGCCTTC

Fgf7(Mus)F	TGGGCACTATATCTCTAGCTTGC
Fgf7(Mus)R	GGGTGCGACAGAACAGTCT
Hgf(Mus)F	ACTTCTGCCGGTCCTGTTG
Hgf(Mus)R	CCCCTGTTCCTGATACACCT
Egf(Mus)F	AGAGCATCTCTCGGATTGACC
Egf(Mus)R	CCCGTTAAGGAAAACTCTTAGCA
Vegfa(Mus)F	TCACGGAGGCAGAGAAAAGAG
Vegfa(Mus)R	CACCGATCTGGGAGAGAGAGA
Tgfβ(Mus)F	CTCCCGTGGCTTCTAGTGC
Tgfβ(Mus)R	GCCTTAGTTTGGACAGGATCTG
Thbs1(Mus)F	GGGGAGATAACGGTGTGTTTG
Thbs1(Mus)R	CGGGGATCAGGTTGGCATT
Bmp4(Mus)F	ATTCCTGGTAACCGAATGCTG
Bmp4(Mus)R	CCGGTCTCAGGTATCAAACTAGC
Bmp6(Mus)F	GCGGGAGATGCAAAAGGAGAT
Bmp6(Mus)R	ATTGGACAGGGCGTTGTAGAG
Wnt7b(Mus)F	CGCTACGGCATCGACTTTTCT
Wnt7b(Mus)R	TCTGCCCGCCTCATTGTTG
